# The midlife health of only children: chronic disease indicators and biomarkers by sibship size in three nationally representative UK cohorts

**DOI:** 10.1093/ije/dyae119

**Published:** 2024-09-03

**Authors:** Jenny Chanfreau, Katherine Keenan, Kieron Barclay, Alice Goisis

**Affiliations:** University of Sussex, Brighton, UK; University College London, London, UK; University of St Andrews, St Andrews, UK; Stockholm University, Stockholm, Sweden; University College London, London, UK

**Keywords:** Only child, sibship size, British birth cohort studies, cardiovascular disease, biomarkers, general health, cancer

## Abstract

**Background:**

Despite persistent concerns about only children’s disadvantage relative to individuals with siblings, existing health-related evidence is inconsistent. Recent evidence from Nordic countries about only children having poorer health outcomes may not apply elsewhere because selection processes differ across contexts. We investigate the midlife health of only children in the UK where one-child families tend to be socio-economically advantaged relative to large families.

**Methods:**

Using the 1946, 1958 and 1970 British birth cohort studies, we examine various biomarkers and self-reported measures of chronic disease by sibship size when respondents are aged in their mid-40s, mid-50s and mid-60s. We estimate separate linear probability models for each cohort, age and outcome, adjusting for childhood and early adulthood circumstances.

**Results:**

We found no evidence of only children differing from those with one, two or three or more siblings, at any age, in any of the cohorts, on: heart problems, hypertension, high triglycerides, high glycated haemoglobin or high C-reactive protein. However, compared with only children, the probability for cancer (0.019, 95% confidence interval [CI]: 0.002, 0.035; age 46/1970) and poor general health (0.060, CI: 0.015, 0.127; age 55/1958; and 0.110, CI: 0.052, 0.168; age 63/1946) was higher among those with three or more siblings.

**Conclusions:**

There is no consistent pattern of only child health disadvantage for midlife chronic disease outcomes across ages or cohorts in the UK. Research should focus on better understanding how sibship size differentials are contingent on context.

Key Messages

- There is limited and mixed evidence on only children's adult health from previous studies.
- Using a range of self-reported and biomarker measures of chronic disease in midlife, we find no consistent evidence of only children’ disadvantage in the UK.
- The finding points to the need for research to focus on better understanding how sibship size differentials in outcomes are contingent on context.

## Introduction

According to persistent stereotypes, only children (individuals without siblings) are less socially competent, more self-centred and more anxious.^1^^,^^2^ These ideas endure despite empirical evidence consistently showing that, on average, only children either do not differ from those with siblings, or even outperform individuals from large families, on cognitive, educational and socio-economic outcomes.^1–5^ Existing evidence is primarily based on outcomes measured during childhood. In contrast, the evidence on outcomes in adulthood, and in particular health, is both sparse and inconsistent. The limited studies on morbidity and mortality by sibship size suggest important variations across contexts.^6–14^

On the one hand, research suggests higher risk of adverse health outcomes among only children. Two recent studies using Swedish national register data have found young men without siblings are more likely to have a body mass index (BMI) categorized as overweight or obese, and lower physical fitness, than individuals with siblings,^6^ and that adult only children have higher risk of cardiovascular disease (but not coronary heart disease) compared with individuals with siblings.^7^ A systematic review and meta-analysis found only children were more likely to be classified as overweight or obese across a range of contexts (the review covered studies from Europe, Asia and the Americas, predominantly focused on adolescence).^8^ A Finnish study found adult only children to have higher risk of hypertension than those with two or more siblings, higher risk of BMI above the obesity threshold than those with one sibling (adjusted for age and sex) and higher risk of high triglyceride among men who were only children than men with two or more siblings.^9^ Mortality risk has also been found to be higher in Sweden among only children (based on national health register data), compared with those with siblings, including mortality attributable to circulatory problems,^6^^,^^7^^,^^10^ and one study of Scottish university graduates found that men without siblings had higher respiratory disease mortality.^11^

On the other hand, the same Scottish study found no association with all-cause mortality nor mortality attributable to coronary heart disease, stroke or stomach cancer,^11^ while another study also using Scottish data found that men who grew up as only children had a *lower* risk of stroke mortality and lung cancer mortality than men with siblings.^12^ A cross-cohort longitudinal analysis found that, in the UK, the difference in BMI between only children and those with siblings is substantively small and limited to childhood,^13^ and the study of only children in Finland cited above found that they did not differ from those with siblings in terms of risk of type II diabetes, prediabetes, high cholesterol or metabolic syndrome.^9^ Finally, a study of German adolescents concluded that despite finding higher blood pressure among only children than those with siblings, the small magnitude of the difference indicated the clinical implications for this age group was limited or negligible.^14^

These inconsistencies may stem from differential selection pathways into being an only-child family influencing only children’s outcomes.^4^^,^^15^^,^^16^ Cross-national analysis has found a positive correlation between the prevalence of one-child families and the socio-demographic characteristics of this group; they tend to be socio-economically advantaged in countries where small families are more prevalent (e.g. Spain, Italy and Greece).^16^ Whilst consistent evidence of adverse health outcomes for only children has primarily been observed in the low prevalence context of Sweden,^16^ the negative association observed there could be confounded by (unobserved) disadvantage,^6^ and might not be generalizable to other countries. Conversely, although research from elsewhere suggests that patterns of health outcomes by sibship size may differ in other contexts, the evidence is scarce, mixed and has analysed a limited set of outcomes.

The objective of this study was to examine a range of direct health outcomes at different time points in mid-adulthood by sibship size, using data from the 1946, 1958 and 1970 British birth cohort studies. Complemented with a measure of self-reported general health, we focus on indicators of cardiovascular problems and cancer in midlife because these chronic diseases are leading causes of high burden of morbidity and mortality in this age group.^17^^,^^18^ Prevention and treatment of these chronic diseases is a key policy objective across high income countries.

Our contribution to the literature is 2-fold. First, we focus on the UK, a context where existing evidence on only children’s health and mortality in adulthood is limited but indicative of different patterns from those found in the Nordic context.^11–13^ Despite a strong family size norm of two or more children, in terms of socio-demographic profiles UK only children tend to be less advantaged than those with one or two siblings and at an advantage relative to individuals from larger families.^4^ The UK context therefore provides an opportunity to place the currently mixed evidence on health outcomes by sibship size in conversation with the wider literature on only children’s circumstances and outcomes differing cross-nationally and over time.

Second, we examine several indicators of chronic disease, both biomarkers and self-reported measures, that are correlated with mortality. Comparing cohorts born 12 years apart allows us to study outcomes at a greater range of ages, use the different datasets to test whether any observed differences by sibship size replicate, and attend to possible indications of change across the cohorts. Analysis of the same datasets has shown that, relative to the earlier cohorts, in the 1970 cohort only children are more likely than those with siblings to have experienced parental separation (which is often associated with disadvantage),^4^ suggesting that the health outcomes of only children in this cohort could differ due to compositional change of the only child group. In addition to potential drivers of selection into being an only child, such as parental socio-economic status (SES) and divorce, these longitudinal studies include measures of childhood circumstances that may affect socio-economic circumstances and health behaviours in adulthood, in turn affecting midlife health risk factors and outcomes. Our analytic approach therefore allows us to make a comprehensive assessment of only children’s midlife health relative to other sibship size groups in the UK context, as we examine our findings for consistent patterns of health by sibship size either for a specific outcome across ages and cohorts, or within a specific cohort across outcomes and ages.

## Methods

### Data

We use data from three British cohort studies which follow individuals born in 1946, 1958 and 1970 in England, Scotland and Wales. We analyse a range of both objectively measured and self-reported indicators of cardiovascular health, cancer and self-assessed general health in midlife (mid-40s to mid-60s). The National Survey of Health and Development^19^^,^^20^ has followed a subsample of the individuals born in a specific week in 1946 (5362 of the initially surveyed 13 687 births) and the 1958 National Child Development Study^21^ and the 1970 British Cohort Study^22^ follow the approximately 17 000 people born in a particular week in 1958 and 1970, respectively. Across these different cohorts we observe health outcomes when the cohort members (CMs) were in their mid-40s (1946 cohort: age 43; 1958 cohort: ages 44/46; 1970 cohort: age 46), mid-50s (1946 cohort: age 53; 1958 cohort: age 55) and mid-60s (1946 cohort: age 63).

### Health outcomes

We analyse a combination of objective biomarker measures and self-reported health assessments, including a mixture of disease diagnoses and markers of metabolic dysfunction indicating increased risk of chronic disease. For all outcomes we use a binary indicator of a diagnosis or elevated risk of health problems.

The biomarkers analysed are blood pressure, triglycerides, glycated haemoglobin (HbA1c) and C-reactive protein (CRP). These biomarkers were measured by nurse interviewers, and we use conventional thresholds to dichotomize readings. Hypertension (blood pressure > 140/90 mmHg) is a risk factor for circulatory disease, high triglyceride levels (>1.7 mmol/l) can be a sign of other conditions that increase the risk of heart disease and stroke, and high CRP (>3.0 g/L) indicate potential inflammation or cardiovascular disease. HbA1c is an index of glucose metabolism over the previous 30–90 days, and high levels (>6%) are indicative of the presence of diabetes mellitus. Nurse-measured blood pressure is not available at age 55 for the 1958 study, and we include respondents’ self-report of having high blood pressure for this age.

The self-reported health assessments analysed include heart problems or diagnosed heart condition, self-reported cancer diagnosis and self-assessed general health as fair/poor/very poor (vs good/very good/excellent). See Supplementary Table S1 (available as Supplementary data at *IJE* online) for details of the measures available at each age.

### Sibship size

We categorize cohort members based on the number of co-resident siblings at age 10/11: Only child, 1, 2, 3+ siblings, using the derivation method outlined by Goisis and Chanfreau.^23^

### Covariates

Given that past research and theories indicate the importance of selection into being an only child for their outcomes, our models control for the following covariates collected during childhood: CM’s sex and birth order; maternal age at CM’s birth; maternal education past compulsory schooling; whether the CM was breastfed; parental social class (register general categories) at birth of CM (in 1946 cohort when CM was aged four years), and an indicator of the co-residence of both biological parents at the age 10/11 sweep as a proxy for parental separation. In addition, we include measures of the CM’s health behaviours and socio-economic position in adulthood sweeps prior to the earliest outcome measures: smoking status; alcohol intake frequency; qualification and occupation. See Supplementary Table S2 (available as Supplementary data at *IJE* online) for covariate categories, ages measured and univariate summary statistics. An in-depth portrait of the socio-economic profile of one-child families compared with other family sizes using these datasets has been detailed elsewhere, showing that one-child families tend to be socio-economically less advantaged than two/three-child families and substantially more advantaged than four-child or larger families.^4^

Given that, as a proportion of the original sample, between 47% and 61% of CMs responded at the ages we analyse, we follow the recommended missing data strategy for these longitudinal studies.^24^^,^^25^ We use multiple imputation with chained equations, creating 50 imputed data sets separately for each cohort and age analysed. See Supplementary Tables S3 and S4 (available as Supplementary data at *IJE* online) for observed and imputed sample sizes at each age analysed, and details on imputation models. Analyses of the 1946 data also applied study design weights to adjust for the sampling procedure.^26^

**Figure 1.**
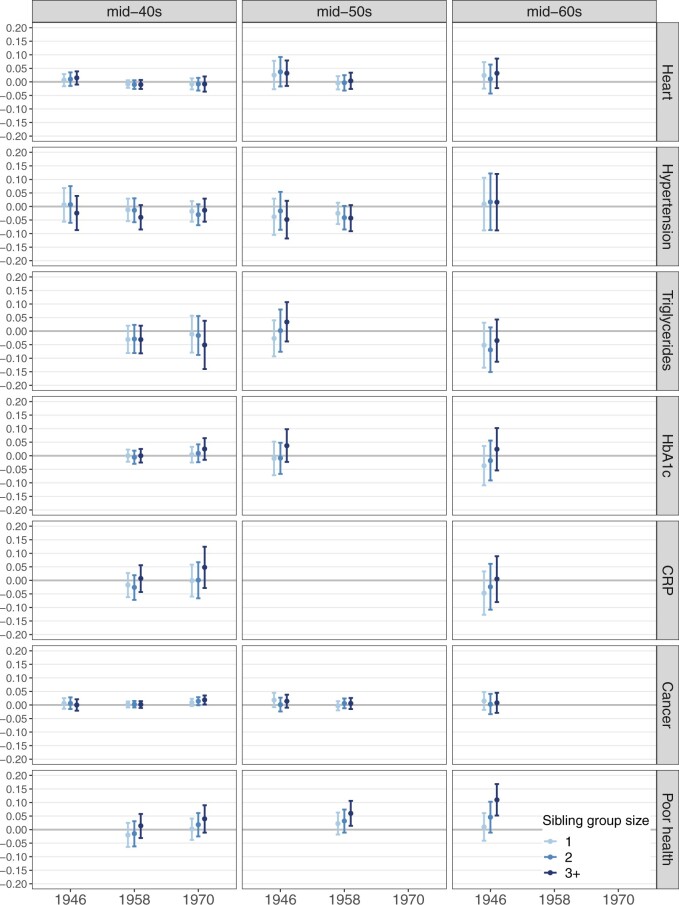
Coefficients for sibship size groups (reference category: only child), from linear probability models with 95% confidence interval. Model specification 2 shown, see Supplementary Table S5 (available as Supplementary data at *IJE* online) for results table. Outcomes are self-reported heart problems, high blood pressure (self-reported at age 55 only), high triglycerides, high Glycated haemoglobin (HbA1c) and high C-reactive protein (CRP), self-reported cancer diagnosis and self-assessed general health as fair/poor/very poor. Models adjust for cohort members’ (CM) sex and birth order, maternal age at CMs’ birth, maternal education, whether the CM was breastfed, paternal social class and parental separation by age 10/11. Regressions run separately for each outcome, age and cohort on multiply imputed data (*M* = 50; see Supplementary Table S3, available as Supplementary data at *IJE* online for details)

We ran linear probability models on the imputed data, fitting separate models for each cohort, age and outcome. The coefficients in linear probability models can be interpreted as the percentage-point change in the probability of the outcome occurring. We ran three model specifications for each outcome: Model 1 (unadjusted) included sibship size only; Model 2 included individual and family covariates collected in childhood; Model 3 additionally controlled for the covariates collected during adulthood. Results did not differ substantively across the model specifications. In the main text, we illustrate the results from Model 2 in Figure 1, displaying coefficients with 95% confidence intervals for sibship groups relative to only children (reference category). For the table of coefficients see Supplementary Table S5 (available as Supplementary data at *IJE* online). All analyses were conducted in Stata 16.

## Results

Figure 1 shows the results from separate linear probability models for each of the indicators of chronic disease, markers of elevated risk of chronic disease and self-reported poor general health, where available, in the mid-40s for all three cohorts, the mid-50s for the 1946 and 1958 cohorts and at age 63 for the 1946 cohort. Overall, the results show limited difference across sibship groups for all measures.

Self-reported heart problems and hypertension recordings (self-reported at age 55) are available at all ages analysed for the three cohorts. Measures of high levels of triglycerides, high HbA1c (indicating diabetes) and high CRP recordings are available for mid-40s ages for the 1958 and 1970 cohort and ages in the mid-50s and mid-60s for the 1946 cohort (CRP only available at age 63). There were no differences by sibship size for any of these outcomes at any age for any cohort. Based on the direction of the point estimates, there is some suggestive evidence of lower probability of hypertension at the mid-40s and mid-50s ages among individuals with siblings compared to only children, although for all coefficients the 95% confidence interval (CI) contains zero.

Self-reported cancer diagnosis is available at all ages analysed for the three cohorts and self-rated poor general health reports are available at ages in the mid-40s for the 1958 and 1970 cohort, in the mid-50s for the 1958 cohort and mid-60s for the 1946 cohort. The prevalence of cancer diagnoses was low at all ages (Supplementary Table S2, available as Supplementary data at *IJE* online) and, as shown in Figure 1, the point estimates and confidence intervals again indicate minimal difference by sibship size. Only at age 46 in the 1970 cohort do individuals with three or more siblings have somewhat elevated probability of reporting cancer (0.019, CI: 0.002, 0.035), compared with only children.

The probability of poor or fair general health did not differ by sibship size in the mid-40s. However, at age 55 in the 1958 cohort and age 63 in the 1946 cohort individuals with three or more siblings when growing up had 6% higher (0.060, CI: 0.015, 0.127, 1958 age 55) and 11% higher (0.110, CI: 0.052, 0.168, 1946 age 63) probability of reporting less than good health, respectively.

As shown in Supplementary Material (Supplementary Tables S3 and S4, available as Supplementary data at *IJE* online), we found some minor differences in mortality and emigration by sibship size but no consistent pattern. Given the small magnitude and lack of systematic pattern of these differences across the ages and cohorts, this is very unlikely to explain the lack of differences in health outcomes by sibship size.

### Robustness checks and additional analyses

As a robustness check, we estimated binary logistic regression models for all outcomes and the results were substantively similar (results available from the authors on request). We also ran analyses on unimputed data and our findings are substantively unchanged (see Supplementary Figure S1, available as Supplementary data at *IJE* online). Finally, we considered whether the general lack of overall difference by sibship size might be due to diversity within the only child group that are masked when analysed as an undifferentiated category. To explore this, we ran models interacting sibship size and parental social class and, separately, interacting sibship size and parental separation. We found no evidence of interactions for any health outcomes at any age (Supplementary Tables S6 and S7, available as Supplementary data at *IJE* online).

## Conclusion

This article investigated the health of only children in the UK in relation to multiple measures of chronic disease, focusing on indicators of cardiovascular problems and cancer which are leading causes of mortality. The evidence in the extant literature is both sparse and mixed for health outcomes by sibship size, possibly because only children’s distinctiveness or lack thereof from those with siblings may be contingent on social context (country and time-period).^4^^,^^16^ We focus on the UK context where one-child families are relatively socially advantaged compared to other contexts, such as Sweden,^6^ where research has highlighted a negative association between lack of siblings and health, and therefore might be at lower risk of poorer health. We investigate multiple midlife chronic disease outcomes at more than one age and for cohorts born twelve years apart and base our conclusions on a general assessment of results across outcomes, ages and cohorts. Overall, in the UK, for these indicators, there is a lack of robust evidence of only child distinctiveness. Despite a small number of coefficients indicating some difference between only children and those raised in a large sibling group, we conclude that there is no consistent pattern of difference either for a specific outcome across ages and cohorts or within a cohort across outcomes and ages. The implication is that demographic trends pointing towards an increasing share of one-child families are not necessarily negative for health over the life course.

There are some limitations to our analysis. First, we do not have objective measures of heart disease or cancer diagnoses from medical records, and we have no biomarker measures at age 55 in the 1958 cohort. Second, the question-wording for the self-reported health outcomes differ across the cohort studies and at different ages, e.g. in relation to the timescale considered, as outlined in detail in Supplementary Table S1 (available as Supplementary data at *IJE* online). These differences may explain the variation in prevalence rates of health problems across ages and cohorts shown in Supplementary Table S2 (available as Supplementary data at *IJE* online). However, given that we conducted regressions separately for each age and cohort this should not affect our findings comparing outcomes by sibship size, reinforced by the lack of consistent difference across both self-reported and measured outcomes. Further, our approach of interpretating the overall pattern across outcomes mitigated against conclusions being influenced by potential biases on a given measure, a risk that might have arisen from basing conclusions on a specific outcome at a specific age. Finally, we do not control for parental investments as a potential explanatory mechanism, capturing this only indirectly by controlling for socio-economic status. Investigation of parental investments as a potential compensatory mechanism may be useful especially in contexts where—unlike the UK—one-child families are particularly socio-economically disadvantaged.

Our findings are consistent with prior analysis of BMI trajectories by sibship size in the UK,^13^ but clearly in contrast with research showing worse health and higher mortality among individuals without siblings in Sweden, compared to those with siblings.^6^^,^^7^^,^^10^ However, in Sweden only children appear to be a more select and disadvantaged group than in the UK, which may explain the difference in findings and the poorer health outcomes for (adult) only children in Sweden remaining notable even after robust adjustment for potential confounders. The overall null finding in this study is important in the context of refuting persistent stereotypes of a universal only child disadvantage attributable to the experience of growing up in a family without siblings. Future research should focus on explaining why different patterns are observed across contexts, such as understanding cross-national variation in the selection into being an only child and in how other potentially important mechanisms such as parental investment vary by sibship size.

## Ethics approval

Ethics approval was not required for this project as the data used had been anonymized by the survey teams prior to being made available for analysis.

## Supplementary Material

dyae119_Supplementary_Data

## Data Availability

The data that support the findings of this study are available to registered users from the UK Data Service (https://ukdataservice.ac.uk/) with End User Licence, and from MRC Unit for Lifelong Health and Ageing at UCL (LHA; https://www.nshd.mrc.ac.uk/) with the permission of LHA.
