# Supplementary material for: The midlife health of only children: chronic disease indicators and biomarkers by sibship size in three nationally representative UK cohorts
Source: Int J Epidemiol. 2024 Sep 3;53(5):dyae119. doi: 10.1093/ije/dyae119 (PMC11371166; doi:10.1093/ije/dyae119)
Supplement: dyae119_Supplementary_Data [file dyae119_supplementary_data.docx]

**The midlife health of only children – Supplementary Materials**

**Supplementary Table S1 Outcome variable measures available at each age**

| **Measure** | **Mid-40s** | **Mid-50s** | **Mid-60s** |
| --- | --- | --- | --- |
| Heart problem (self-reported) | - 1946 (43): “Ever had heart trouble?” - 1958 (46): Derived from ICD-10 codes in responses to “Any long-standing illness, disability or infirmity?" or "Other health problem in the last 12 months" - 1970 (46): “Since the last interview/in the last four years, have you had any of the health problems listed: Heart problems?” | - 1946 (53): If any mentioned in response to “which of these kinds of heart trouble you have had, if any, in the last ten years that is since you were 43 years old?” - 1958 (55): “Since [last interview / five years ago] have you had any of these conditions or health problems: Heart problems?” | - 1946 (53): Since 1999 has a doctor told you that you have had angina?/heart attack (myocardial infarct/coronary thrombosis)?/ any other heart trouble suspected or confirmed? (e.g. valve disease, congenital heart disease or irregular heart beat) |
| Hypertension (>140/90mmHg, or taking anti- hypertensive medication) | - 1946 (43): measured - 1958 (44): measured - 1970 (46): measured | - 1946 (53): measured - 1958 (55): (self-reported) “Since [last interview / five years ago] have you had any of these conditions or health problems: High blood pressure?” | - 1946 (63): measured |
| High triglycerides (>1.7mmol/L) | - 1946 (43): n/a - 1958 (44): measured - 1970 (46): measured | - 1946 (53): measured - 1958 (55): n/a | - 1946 (63): measured |
| High Glycated haemoglobin (HbA1c)  (>6% or >42mmol/mol) | - 1946 (43): n/a - 1958 (44): measured - 1970 (46): measured | - 1946 (53): measured - 1958 (55): n/a | - 1946 (63): measured |
| C-reactive protein (CRP) (>3.0g/L) | - 1946 (43): n/a - 1958 (44): measured - 1970 (46): measured | - 1946 (53): n/a - 1958 (55): n/a | - 1946 (63): measured |
| Cancer  (self-reported) | - 1946 (43): “Have you ever had cancer?” - 1958 (46): Derived from ICD-10 codes in responses to “Any long-standing illness, disability or infirmity?" or "Other health problem in the last 12 months" - 1970 (46): “Since the last interview/in the last four years, have you had any of the health problems listed: Cancer or Leukaemia?” | - 1946 (53): “And in the last ten years (that is since you were 43 years old,) have you had cancer?” - 1958 (55): “Since [last interview / five years ago] have you had any of these conditions or health problems: Cancer or Leukaemia?” | - 1946 (63): “Since 1999 have you been told by a doctor that you have cancer?” |
| General health (self-reported) | - 1946 (43): n/a - 1958 (46): Answered 3, 4 or 5 to “Please think back over the last 12 months about how your health has been. Compared to people of your own age, would you say that your health has on the whole been 1 excellent, 2 good, 3 fair, 4 poor or 5 very poor?” - 1970 (46): Answered 4 or 5 to “In general, would you say your health is… 1 Excellent 2 Very good 3 Good 4 Fair 5 Poor” | - 1946 (53): n/a - 1958 (55): Answered 4 or 5 to “In general, would you say your health is… 1 Excellent 2 Very good 3 Good 4 Fair 5 Poor” | - 1946 (63): Answered 4 or 5 to “How is your health in general? 1 Excellent 2 Very good 3 Good 4 Fair 5 Poor” |

*Notes: Rows and text highlighted in grey denotes self-reported measures. Biomarker measures were collected by trained nurse interviewers and recorded on a continuous scale in the raw data, binary variables were derived by the authors.*

**Supplementary Table S2 Univariate summary statistics of health outcomes, sibship size and covariates**

| **Cohort** | **1946** | **1958** | **1970** | **1946** | **1958** | **1946** |
| --- | --- | --- | --- | --- | --- | --- |
| **Age** | **43** | **44/46** | **46** | **53** | **55** | **63** |
|  | **%** | **%** | **%** | **%** | **%** | **%** |
| **Outcomes** |  |  |  |  |  |  |
| Heart problem | 3.8 | 1.6 | 4.1 | 15.2 | 6.4 | 14.8 |
| Hypertension | 26.1 | 24.3 | 23.6 | 50.3 | 23.3 | 59.0 |
| High triglycerides |  | 47.5 | 40.3 | 55.1 |  | 25.5 |
| High glycated haemoglobin |  | 4.3 | 9.3 | 15.1 |  | 24.9 |
| High C-reactive protein |  | 20.8 | 24.8 |  |  | 38.8 |
| Cancer | 2.0 | 1.1 | 1.7 | 3.5 | 3.7 | 6.3 |
| Poor general health |  |  | 23.6 |  | 22.9 | 18.9 |

| **Cohort** | **1946** | **1958** | **1970** |
| --- | --- | --- | --- |
|  | **%** | **%** | **%** |
| **Sibship size** |  |  |  |
| Only child | 12.9 | 7.2 | 8.6 |
| 1 sibling | 30.0 | 28.9 | 46.8 |
| 2 siblings | 23.5 | 25.0 | 28.1 |
| 3+ siblings | 33.6 | 38.9 | 16.5 |
| **Covariates collected in childhood** |  |  |  |
| Sex: Female | 48.0 | 49.0 | 48.8 |
| Birth order: Firstborn | 39.1 | 37.5 | 42.3 |
| Second order birth | 31.3 | 29.8 | 30.3 |
| Third or higher order birth | 29.5 | 32.7 | 27.4 |
| Mothers age: Under 20 | 2.5 | 5.6 | 10.7 |
| 20-24 | 24.0 | 28.4 | 36.9 |
| 25-29 | 29.7 | 32.3 | 30.1 |
| 30-34 | 26.0 | 20.5 | 14.5 |
| 35-39 | 13.6 | 10.6 | 5.9 |
| 40 and over | 4.2 | 2.5 | 1.9 |
| Ever breastfed | 62.6 | 68.0 | 37.2 |
| Mother stayed in school | 28.3 | 24.6 | 35.6 |
| Parental social class: I | 2.5 | 4.4 | 5.0 |
| II | 10.9 | 12.9 | 13.1 |
| III non-manual | 9.1 | 9.7 | 13.8 |
| III manual | 41.9 | 50.4 | 44.5 |
| VI | 18.8 | 12.4 | 17.1 |
| V | 8.9 | 10.1 | 6.4 |
| n/a, unemployed | 7.9 |  |  |
| Parents separated by age 10/11 | 11.0 | 8.4 | 31.1 |
| **Covariates collected in adulthood** |  |  |  |
| Highest qualification: None | 51.1 | 14.6 | 15.5 |
| CSE 2-5/equiv NVQ1 | 5.2 | 13.5 | 9.3 |
| O Level/equiv NVQ2 | 18.8 | 34.0 | 31.8 |
| A Level/equiv NVQ3 | 10.9 | 13.2 | 13.6 |
| Higher qual NVQ4 | 8.1 | 13.3 | 25.8 |
| Degree/higher NVQ5,6 | 5.9 | 11.5 | 4.0 |
| Social class: Professional | 5.2 | 4.6 | 4.6 |
| Managerial\tech | 20.7 | 34.5 | 34.6 |
| Skilled non-manual | 15.2 | 21.0 | 14.4 |
| Skilled manual | 20.4 | 21.3 | 15.3 |
| Partly skilled | 13.0 | 14.0 | 10.3 |
| Unskilled | 3.3 | 4.7 | 2.0 |
| Not working/Other | 22.2 |  | 18.9 |
| Smoking status: Never | 33.1 | 47.2 | 40.8 |
| Former | 20.5 | 18.2 | 18.1 |
| Current | 46.4 | 34.5 | 41.0 |
| Alcohol frequency: Never |  | 4.6 | 6.3 |
| < Monthly |  | 17.2 | 14.4 |
| 2-3x month |  | 19.6 | 13.7 |
| 1-3x week |  | 46.5 | 53.2 |
| Most days |  | 12.1 | 12.4 |

*Notes: We note that age 55 (1958 cohort) is the only time point when high blood pressure is self-reported, rather than measured. Rates of self-reported high blood pressure tend to be lower than when measured, likely because many are unaware of their hypertension. However, we have no reason to believe that awareness or reporting would systematically differ by sibship size. Maternal age recorded at time of cohort members’ (CM) birth, parental social class (paternal in 1946 and 1957 cohort; highest of two parents in 1970) recorded when CM was age four years (1946) or at time of CM’s birth (1958, 1970). Adulthood covariates collected at ages: highest level of qualification age 31 (1946), 33 (1958), 30 (1970); and occupation age 31 (1946), 42 (1958), 42 (1970); CM’s smoking status age 31 (1946), 33 (1958), 30 (1970); alcohol intake frequency age 33 (1958), 30 (1970).*

**Supplementary Table S3 Analysis sample: Comparison of observed and multiple imputation (MI) sample sizes**

|  | Mid-40s | | | | Mid-50s | | Mid-60s |
| --- | --- | --- | --- | --- | --- | --- | --- |
| Cohort | 1946 | 1958 | 1958 | 1970 | 1946 | 1958 | 1946 |
| Age | 43 | 44 | 46 | 46 | 53 | 55 | 63 |
| Participated in Sweep | 3,262 | 9,377 | 9,534 | 8,581 | 3,035 | 9,137 | 2,662 |
| Non-missing on outcome | 3,186 – 3,262 | 7,667 – 9,227 | 9,529 | 3,421 – 8,580 | 2,561 – 3,035 | 9,012 – 9,025 | 1,289 – 2,474 |
| Non-missing on sibship size | 4,182 | 13,134 | 13,134 | 13,404 | 4,182 | 13,134 | 4,182 |
| Non-missing on all covariates | 2,370 | 6,017 | 6,017 | 6,657 | 2,370 | 6,017 | 2,370 |
| Complete cases | 2,056 | 3,920 | 5,047 | 1,449 | 1,600 | 4,744 | 701 |
| Original sample size ^1^ | 5,362 | 18,555 | 18,555 | 18,031 | 5,362 | 18,555 | 5,362 |
| Emigrated or died ^2^ | 628 | 2,522 | 2,595 | 1,446 | 615 | 2,945 | 608 |
| MI sample ^3^ | 4,734 | 16,036 | 15,963 | 16,585 | 4,747 | 15,613 | 4,754 |

*Notes: ^1^ Present in the outcome file and non-missing on cohort member (CM) sex. ^2^ Emigration or cohort member death not available for 1946 cohort; we exclude individuals who have not participated in the study since age 15 up to and including the outcome sweep. ^3^ 50 imputed datasets.*

*As can be seen from the table much of the reduction of in the original sample size is due to the combination of sweep non-response at the ages analysed in midlife and the number of cases with non-missing information on all covariates. The latter is due to the fact that these were recorded at a variety of different ages (in childhood, and different ages earlier in adulthood), requiring CMs to have taken part in all of those sweeps. A comparison of the number of respondents in a given sweep and the number that responded to the health indicators shows that for these long-running studies, missingness is primarily driven by attrition (sweep non-response) rather than item non-response. Missing data best-practice guidelines suggest using techniques such as multiple imputation (MI) to improve the plausibility of the missing at random assumption (given the covariates and auxiliary variables),^[[1]](#footnote-1)^ and one strength of the cohort data is the extensive availability of variables measured prior to non-response that can be included in the imputation models. The aim of using MI is to yield valid analysis results by restoring sample representativeness of the target population, which for our analysis is all people born in the cohort year and alive and resident in Great Britain at the age we analyse. Prior to imputation, we therefore exclude those who have emigrated or died (in the 1958 and 1970 cohorts where this detail is available to us).*

*Analysis of emigration and death by sibship size showed some minor differences but did not reveal systematic patterns across the ages and both cohorts (Supplementary Table S4). In the 1970 cohort, there is some indication of somewhat elevated mortality by age 46 among only children (3.4% compared with 2.0-2.9% among those with siblings). On the other hand, in the 1958 cohort those with 3+ siblings have the highest mortality at age 55 and lowest likelihood of having emigrated by age 44.* *However, there is some indication that participation at a given sweep may differ* *systematically by sibship size**, with participation generally lower among CMs with 3+ siblings and to a lesser extent only children (Supplementary Table S4), suggesting that an approach such as MI may be needed to correct for any potential bias introduced by differential response.*

*Imputation model specifications: We dropped cohort members known to have died or emigrated by the outcome sweep (1958 & 1970) or not participated between age 16 & the outcome sweep (1946) and cohort members with missing information on sex prior to imputation. We included auxiliary variables that are both significantly related to missingness (i.e. non-response in the sweep of data analysed) and related to at least one of the outcome variable in the substantive model(s), and also a measure of BMI at an earlier adult age as an auxiliary as this was associated with a number of the outcomes. We ran separate imputation models for each cohort and age analysed, including all outcome variables for that age, all auxiliary variables and all analysis covariates in the imputation model. Prior to analysis, we checked that the imputed values for variables included in the analysis model were plausible by comparing summary statistics before and after imputation. Auxiliary variables included in in each imputation model:*

- *1946 - Ages 43, 53 & 63: Whether father stayed in school past minimum leaving age, Ownership of house (age 36), Age 11 Cognitive test score, Education tests Mathematics at15 years raw score*
- *1958 - Age 44: Age father left school, Birth sweep – accommodation number of persons per room, Age 7 copy design, Age 11 Total score on general ability test, Age 11 Z score behavioural problems, Age 16 Z score behavioural problems, Age 16 school attendance, Age 16 How long since drank alcohol, Age 33 Telephone in home, Age 33 Physical effort in job.*
- *1958 - Age 46: Age father left school, Birth sweep – accommodation number of persons per room, Age 7 copy design, Age 11 Total score on general ability test, Age 11 Z score behavioural problems, Age 16 Z score behavioural problems, Age 16 school attendance Voted in 1979 GE, Age 33 Telephone in home, Age 33 Housing tenure, Legal marital status age 23, Age 33 Physical effort in job, Marital status (hist) 42, Number of own children 42*
- *1958 - Age 55: Age father left school, Birth sweep – accommodation number of persons per room, Age 7 copy design, Age 11 Total score on general ability test, Age 11 Z score behavioural problems, Age 16 Z score behavioural problems, Age 16 school attendance, Voted in 1979 GE, Legal marital status age 23, Age 33 Telephone in home, Age 33 Housing tenure, Age 33, Physical effort in job, Marital status (hist) 42, Number of own children 42, Legal marital status age 46, Age 50 Pensions: employer provided Pension*
- *1970 - Age 46: Age father left school, Age 5 copy design, Age 10 behavioural problems, Age 10 BAS word similarity sum, Age 16 reading, Age 16 Rutter score, Age 16 special education, Age 26 Outcome, Age 30 Outcome, Age 30 voted 97, Age 34 Outcome, Age 30 mar stat, Age 30 tenure, Marital status 42, Smoking status 42, Number of own children 42, Alcohol frequency 42*

**Supplementary Table S4 Response, emigration and mortality rates by cohort and age**

|  | **1946** | **1958** | **1958** | **1970** | **1946** | **1958** | **1946** |
| --- | --- | --- | --- | --- | --- | --- | --- |
|  | **43** | **44** | **46** | **46** | **53** | **55** | **63** |
| **Responded** |  |  |  |  |  |  |  |
| Only child | 67.4 | 58.8 | 59.5 | 53.2 | 62.9 | 58.4 | 56.2 |
| 1 sibling | 69.7 | 61.0 | 62.8 | 57.7 | 64.5 | 60.1 | 59.1 |
| 2 siblings | 68.3 | 60.4 | 62.0 | 55.5 | 66.1 | 60.2 | 56.6 |
| 3+ siblings | 67.6 | 55.9 | 55.8 | 46.3 | 61.3 | 51.9 | 52.1 |
| **Emigrated** | n/a |  |  |  | n/a |  | n/a |
| Only child |  | 3.4 | 3.0 | 3.0 |  | 2.9 |  |
| 1 sibling |  | 4.0 | 3.8 | 2.5 |  | 3.8 |  |
| 2 siblings |  | 4.4 | 4.1 | 2.9 |  | 3.9 |  |
| 3+ siblings |  | 3.0 | 3.3 | 2.6 |  | 3.6 |  |
| **Dead** | n/a |  |  |  | n/a |  | n/a |
| Only child |  | 2.3 | 2.3 | 3.4 |  | 4.8 |  |
| 1 sibling |  | 2.1 | 2.4 | 2.3 |  | 3.8 |  |
| 2 siblings |  | 2.7 | 2.9 | 2.0 |  | 4.4 |  |
| 3+ siblings |  | 2.9 | 3.1 | 2.9 |  | 5.8 |  |

**Supplementary Table S5 Summary of regression results: Coefficients for sibship size groups (reference category: Only Child) for each health outcome, separately by cohort and age**

|  | **Sibship size** | | | | | | **Model 1** | | | | **Model 2** | | | **Model 3** | | |
| --- | --- | --- | --- | --- | --- | --- | --- | --- | --- | --- | --- | --- | --- | --- | --- | --- |
|  |  |  |  |  |  |  | **Coeff.** | | **se** | **p-value** | **Coeff.** | **se** | **p-value** | **Coeff.** | **se** | **p-value** |
| **1946 age 43** | |  | | | | |  | |  |  |  |  |  |  |  |  |
| Heart | 1 sib | | | | | | 0.006 | | 0.012 | 0.586 | 0.007 | 0.012 | 0.566 | 0.008 | 0.011 | 0.499 |
|  | 2 sibs | | | | | | 0.010 | | 0.013 | 0.436 | 0.010 | 0.013 | 0.448 | 0.012 | 0.013 | 0.362 |
|  | 3+ | | | | | | 0.017 | | 0.012 | 0.176 | 0.015 | 0.013 | 0.249 | 0.017 | 0.013 | 0.186 |
| BP | 1 sib | | | | | | 0.007 | | 0.032 | 0.835 | 0.006 | 0.032 | 0.854 | 0.005 | 0.032 | 0.870 |
|  | 2 sibs | | | | | | 0.010 | | 0.035 | 0.775 | 0.007 | 0.034 | 0.837 | 0.002 | 0.035 | 0.959 |
|  | 3+ | | | | | | -0.014 | | 0.032 | 0.669 | -0.024 | 0.032 | 0.453 | -0.031 | 0.033 | 0.339 |
| Cancer | 1 sib | | | | | | 0.003 | | 0.010 | 0.742 | 0.006 | 0.010 | 0.584 | 0.006 | 0.010 | 0.555 |
|  | 2 sibs | | | | | | 0.004 | | 0.011 | 0.696 | 0.006 | 0.011 | 0.581 | 0.006 | 0.011 | 0.607 |
|  | 3+ | | | | | | -0.000 | | 0.011 | 0.965 | 0.000 | 0.011 | 0.992 | -0.001 | 0.011 | 0.930 |
| **1958 age 44/46** | | | | | |  | |  |  |  |  |  |  |  |  |  |
| Heart | 1 sib | | | | | | -0.007 | | 0.007 | 0.319 | -0.008 | 0.007 | 0.275 | -0.008 | 0.007 | 0.286 |
|  | 2 sibs | | | | | | -0.007 | | 0.007 | 0.359 | -0.010 | 0.008 | 0.214 | -0.010 | 0.008 | 0.207 |
|  | 3+ | | | | | | -0.003 | | 0.007 | 0.697 | -0.010 | 0.008 | 0.249 | -0.012 | 0.008 | 0.161 |
| BP | 1 sib | | | | | | -0.013 | | 0.020 | 0.517 | -0.012 | 0.021 | 0.562 | -0.012 | 0.021 | 0.565 |
|  | 2 sibs | | | | | | -0.010 | | 0.021 | 0.637 | -0.014 | 0.022 | 0.547 | -0.015 | 0.022 | 0.513 |
|  | 3+ | | | | | | -0.024 | | 0.020 | 0.236 | -0.040 | 0.023 | 0.084 | -0.042 | 0.023 | 0.063 |
| Triglyc. | 1 sib | | | | | | -0.028 | | 0.025 | 0.261 | -0.031 | 0.026 | 0.236 | -0.028 | 0.026 | 0.274 |
|  | 2 sibs | | | | | | -0.020 | | 0.025 | 0.423 | -0.029 | 0.027 | 0.269 | -0.032 | 0.026 | 0.232 |
|  | 3+ | | | | | | -0.002 | | 0.023 | 0.946 | -0.031 | 0.026 | 0.236 | -0.042 | 0.026 | 0.100 |
| HbA1c | 1 sib | | | | | | -0.002 | | 0.011 | 0.879 | 0.000 | 0.011 | 0.983 | 0.001 | 0.011 | 0.953 |
|  | 2 sibs | | | | | | -0.006 | | 0.012 | 0.587 | -0.006 | 0.012 | 0.645 | -0.007 | 0.012 | 0.594 |
|  | 3+ | | | | | | 0.003 | | 0.011 | 0.775 | 0.000 | 0.013 | 0.977 | -0.004 | 0.013 | 0.758 |
| CRP | 1 sib | | | | | | -0.025 | | 0.022 | 0.254 | -0.017 | 0.023 | 0.444 | -0.016 | 0.023 | 0.489 |
|  | 2 sibs | | | | | | -0.029 | | 0.022 | 0.185 | -0.026 | 0.023 | 0.257 | -0.028 | 0.023 | 0.221 |
|  | 3+ | | | | | | 0.016 | | 0.021 | 0.458 | 0.007 | 0.025 | 0.789 | -0.007 | 0.025 | 0.772 |
| Cancer | 1 sib | | | | | | 0.001 | | 0.005 | 0.787 | 0.002 | 0.006 | 0.711 | 0.002 | 0.006 | 0.717 |
|  | 2 sibs | | | | | | 0.002 | | 0.005 | 0.685 | 0.003 | 0.006 | 0.642 | 0.003 | 0.006 | 0.657 |
|  | 3+ | | | | | | 0.002 | | 0.005 | 0.780 | 0.002 | 0.006 | 0.793 | 0.002 | 0.006 | 0.800 |
| General | 1 sib | | | | | | -0.019 | | 0.021 | 0.365 | -0.020 | 0.023 | 0.382 | -0.018 | 0.022 | 0.409 |
| health | 2 sibs | | | | | | 0.004 | | 0.022 | 0.859 | -0.015 | 0.024 | 0.521 | -0.019 | 0.023 | 0.410 |
|  | 3+ | | | | | | 0.066 | | 0.019 | 0.001 | 0.014 | 0.023 | 0.544 | -0.011 | 0.022 | 0.616 |
| **1970 age 46** | | | | |  | |  | |  |  |  |  |  |  |  |  |
| Heart | 1 sib | | | | | | -0.007 | | 0.010 | 0.493 | -0.008 | 0.011 | 0.457 | -0.007 | 0.010 | 0.497 |
|  | 2 sibs | | | | | | -0.004 | | 0.011 | 0.694 | -0.008 | 0.012 | 0.480 | -0.010 | 0.012 | 0.386 |
|  | 3+ | | | | | | -0.002 | | 0.012 | 0.869 | -0.008 | 0.014 | 0.554 | -0.015 | 0.014 | 0.277 |
| BP | 1 sib | | | | | | -0.024 | | 0.019 | 0.214 | -0.018 | 0.019 | 0.359 | -0.019 | 0.019 | 0.326 |
|  | 2 sibs | | | | | | -0.024 | | 0.019 | 0.199 | -0.030 | 0.020 | 0.122 | -0.035 | 0.020 | 0.077 |
|  | 3+ | | | | | | 0.004 | | 0.020 | 0.839 | -0.014 | 0.022 | 0.521 | -0.024 | 0.022 | 0.277 |
| Triglyc. | 1 sib | | | | | | 0.003 | | 0.034 | 0.925 | -0.011 | 0.035 | 0.751 | -0.011 | 0.035 | 0.748 |
|  | 2 sibs | | | | | | 0.017 | | 0.035 | 0.632 | -0.016 | 0.037 | 0.667 | -0.022 | 0.036 | 0.552 |
|  | 3+ | | | | | | -0.010 | | 0.039 | 0.808 | -0.051 | 0.046 | 0.261 | -0.064 | 0.045 | 0.157 |
| HbA1c | 1 sib | | | | | | -0.003 | | 0.015 | 0.855 | 0.004 | 0.015 | 0.785 | 0.005 | 0.014 | 0.722 |
|  | 2 sibs | | | | | | 0.009 | | 0.016 | 0.575 | 0.009 | 0.017 | 0.594 | 0.004 | 0.017 | 0.820 |
|  | 3+ | | | | | | 0.032 | | 0.019 | 0.097 | 0.025 | 0.020 | 0.221 | 0.008 | 0.021 | 0.686 |
| CRP | 1 sib | | | | | | -0.024 | | 0.030 | 0.430 | -0.001 | 0.030 | 0.986 | 0.002 | 0.030 | 0.946 |
|  | 2 sibs | | | | | | -0.016 | | 0.032 | 0.621 | 0.001 | 0.034 | 0.981 | -0.004 | 0.034 | 0.910 |
|  | 3+ | | | | | | 0.044 | | 0.031 | 0.161 | 0.048 | 0.039 | 0.215 | 0.031 | 0.039 | 0.426 |
| Cancer | 1 sib | | | | | | 0.008 | | 0.006 | 0.199 | 0.009 | 0.007 | 0.158 | 0.009 | 0.007 | 0.165 |
|  | 2 sibs | | | | | | 0.009 | | 0.006 | 0.137 | 0.014 | 0.008 | 0.063 | 0.013 | 0.007 | 0.073 |
|  | 3+ | | | | | | 0.012 | | 0.007 | 0.091 | 0.019 | 0.008 | 0.026 | 0.017 | 0.008 | 0.034 |
| General | 1 sib | | | | | | -0.004 | | 0.020 | 0.836 | 0.002 | 0.020 | 0.932 | 0.008 | 0.020 | 0.690 |
| health | 2 sibs | | | | | | 0.033 | | 0.020 | 0.096 | 0.018 | 0.022 | 0.412 | 0.010 | 0.021 | 0.629 |
|  | 3+ | | | | | | 0.077 | | 0.022 | 0.000 | 0.040 | 0.026 | 0.123 | 0.005 | 0.025 | 0.842 |
| **1946 age 53** | | | |  | | |  | |  |  |  |  |  |  |  |  |
| Heart | 1 sib | | | | | | 0.024 | | 0.027 | 0.373 | 0.025 | 0.027 | 0.343 | 0.026 | 0.027 | 0.337 |
|  | 2 sibs | | | | | | 0.036 | | 0.028 | 0.204 | 0.037 | 0.028 | 0.181 | 0.037 | 0.028 | 0.191 |
|  | 3+ | | | | | | 0.033 | | 0.024 | 0.166 | 0.032 | 0.024 | 0.182 | 0.031 | 0.025 | 0.222 |
| BP | 1 sib | | | | | | -0.039 | | 0.035 | 0.265 | -0.038 | 0.034 | 0.271 | -0.044 | 0.034 | 0.197 |
|  | 2 sibs | | | | | | -0.014 | | 0.036 | 0.703 | -0.016 | 0.036 | 0.655 | -0.027 | 0.036 | 0.454 |
|  | 3+ | | | | | | -0.036 | | 0.035 | 0.310 | -0.048 | 0.035 | 0.171 | -0.065 | 0.036 | 0.075 |
| Triglyc. | 1 sib | | | | | | -0.022 | | 0.035 | 0.524 | -0.027 | 0.034 | 0.436 | -0.031 | 0.034 | 0.359 |
|  | 2 sibs | | | | | | 0.007 | | 0.041 | 0.859 | 0.002 | 0.040 | 0.960 | -0.011 | 0.040 | 0.778 |
|  | 3+ | | | | | | 0.046 | | 0.037 | 0.217 | 0.034 | 0.037 | 0.351 | 0.016 | 0.037 | 0.676 |
| HbA1c | 1 sib | | | | | | -0.010 | | 0.032 | 0.754 | -0.010 | 0.032 | 0.751 | -0.014 | 0.031 | 0.657 |
|  | 2 sibs | | | | | | -0.009 | | 0.030 | 0.754 | -0.009 | 0.029 | 0.752 | -0.021 | 0.030 | 0.474 |
|  | 3+ | | | | | | 0.038 | | 0.031 | 0.217 | 0.037 | 0.031 | 0.227 | 0.017 | 0.032 | 0.595 |
| Cancer | 1 sib | | | | | | 0.018 | | 0.014 | 0.184 | 0.018 | 0.014 | 0.175 | 0.019 | 0.014 | 0.166 |
|  | 2 sibs | | | | | | 0.001 | | 0.013 | 0.968 | 0.001 | 0.013 | 0.926 | 0.002 | 0.013 | 0.901 |
|  | 3+ | | | | | | 0.012 | | 0.012 | 0.320 | 0.014 | 0.012 | 0.254 | 0.015 | 0.013 | 0.232 |
| **1958 age 55** | | | |  | | |  | |  |  |  |  |  |  |  |  |
| Heart | 1 sib | | | | | | -0.001 | | 0.012 | 0.935 | -0.004 | 0.013 | 0.779 | -0.003 | 0.013 | 0.811 |
|  | 2 sibs | | | | | | 0.005 | | 0.013 | 0.713 | -0.003 | 0.014 | 0.821 | -0.004 | 0.014 | 0.803 |
|  | 3+ | | | | | | 0.020 | | 0.012 | 0.086 | 0.004 | 0.015 | 0.812 | -0.001 | 0.015 | 0.961 |
| BP | 1 sib | | | | | | -0.025 | | 0.019 | 0.189 | -0.025 | 0.020 | 0.210 | -0.024 | 0.020 | 0.232 |
|  | 2 sibs | | | | | | -0.032 | | 0.020 | 0.104 | -0.041 | 0.022 | 0.064 | -0.042 | 0.022 | 0.061 |
|  | 3+ | | | | | | -0.019 | | 0.020 | 0.334 | -0.043 | 0.025 | 0.078 | -0.052 | 0.025 | 0.037 |
| Cancer | 1 sib | | | | | | -0.003 | | 0.008 | 0.730 | -0.003 | 0.009 | 0.730 | -0.003 | 0.009 | 0.728 |
|  | 2 sibs | | | | | | 0.007 | | 0.008 | 0.400 | 0.006 | 0.009 | 0.517 | 0.006 | 0.009 | 0.492 |
|  | 3+ | | | | | | 0.007 | | 0.008 | 0.406 | 0.006 | 0.010 | 0.583 | 0.006 | 0.010 | 0.570 |
| General | 1 sib | | | | | | 0.029 | | 0.020 | 0.145 | 0.022 | 0.021 | 0.278 | 0.025 | 0.020 | 0.209 |
| health | 2 sibs | | | | | | 0.051 | | 0.020 | 0.010 | 0.032 | 0.022 | 0.146 | 0.029 | 0.021 | 0.173 |
|  | 3+ | | | | | | 0.102 | | 0.019 | 0.000 | 0.060 | 0.023 | 0.010 | 0.034 | 0.023 | 0.150 |
| **1946 age 63** | | |  | | | |  | |  |  |  |  |  |  |  |  |
| Heart | 1 sib | | | | | | 0.025 | | 0.025 | 0.311 | 0.024 | 0.025 | 0.333 | 0.023 | 0.025 | 0.359 |
|  | 2 sibs | | | | | | 0.012 | | 0.027 | 0.650 | 0.011 | 0.027 | 0.691 | 0.003 | 0.027 | 0.919 |
|  | 3+ | | | | | | 0.036 | | 0.028 | 0.211 | 0.032 | 0.028 | 0.260 | 0.018 | 0.029 | 0.541 |
| BP | 1 sib | | | | | | 0.008 | | 0.050 | 0.872 | 0.009 | 0.049 | 0.856 | 0.003 | 0.049 | 0.953 |
|  | 2 sibs | | | | | | 0.020 | | 0.054 | 0.709 | 0.017 | 0.053 | 0.744 | -0.000 | 0.053 | 0.997 |
|  | 3+ | | | | | | 0.028 | | 0.053 | 0.591 | 0.016 | 0.053 | 0.762 | -0.007 | 0.053 | 0.897 |
| Triglyc. | 1 sib | | | | | | -0.054 | | 0.042 | 0.202 | -0.052 | 0.042 | 0.223 | -0.061 | 0.043 | 0.152 |
|  | 2 sibs | | | | | | -0.069 | | 0.042 | 0.107 | -0.069 | 0.042 | 0.104 | -0.087 | 0.043 | 0.042 |
|  | 3+ | | | | | | -0.024 | | 0.041 | 0.558 | -0.035 | 0.040 | 0.382 | -0.062 | 0.042 | 0.136 |
| HbA1c | 1 sib | | | | | | -0.039 | | 0.037 | 0.292 | -0.037 | 0.037 | 0.322 | -0.041 | 0.038 | 0.281 |
|  | 2 sibs | | | | | | -0.018 | | 0.037 | 0.634 | -0.018 | 0.038 | 0.638 | -0.033 | 0.039 | 0.400 |
|  | 3+ | | | | | | 0.030 | | 0.039 | 0.447 | 0.024 | 0.040 | 0.547 | 0.001 | 0.041 | 0.987 |
| CRP | 1 sib | | | | | | -0.051 | | 0.041 | 0.213 | -0.047 | 0.041 | 0.252 | -0.057 | 0.041 | 0.165 |
|  | 2 sibs | | | | | | -0.027 | | 0.043 | 0.538 | -0.024 | 0.043 | 0.584 | -0.052 | 0.043 | 0.231 |
|  | 3+ | | | | | | 0.016 | | 0.042 | 0.703 | 0.005 | 0.043 | 0.912 | -0.042 | 0.044 | 0.338 |
| Cancer | 1 sib | | | | | | 0.014 | | 0.017 | 0.399 | 0.015 | 0.017 | 0.375 | 0.017 | 0.017 | 0.319 |
|  | 2 sibs | | | | | | 0.003 | | 0.019 | 0.879 | 0.003 | 0.019 | 0.858 | 0.006 | 0.019 | 0.751 |
|  | 3+ | | | | | | 0.005 | | 0.019 | 0.807 | 0.008 | 0.019 | 0.660 | 0.012 | 0.020 | 0.526 |
| General | 1 sib | | | | | | 0.005 | | 0.026 | 0.849 | 0.010 | 0.026 | 0.707 | 0.000 | 0.026 | 0.999 |
| health | 2 sibs | | | | | | 0.044 | | 0.029 | 0.126 | 0.046 | 0.029 | 0.111 | 0.023 | 0.029 | 0.428 |
|  | 3+ | | | | | | 0.121 | | 0.029 | 0.000 | 0.110 | 0.030 | 0.000 | 0.074 | 0.030 | 0.014 |

*Note: Imputed data (M=50). Observations at each cohort and age: 4,734 (1946, age 43); 15,963 (1958, age 44); 16,036 (1958, age 46); 16,585 (1970, age 46); 4,747 (1946, age 53); 15,613 (1958, age 55); 4,754 (1946, age 63). Model 1 is unadjusted. Model 2 adjusts for cohort members’ (CM) sex and birth order, maternal age at CM’s birth, maternal education, whether the CM was breastfed, paternal social class and parental separation by age 10/11. Model 3 adjusts for all covariates listed for model 2 and CM’s smoking status; alcohol intake frequency; highest level of qualification; and occupation.*

**Supplementary Figure S1 Complete cases analysis: Coefficients for sibship size groups (reference category: Only Child), from linear probability models with 95% confidence intervals**

*
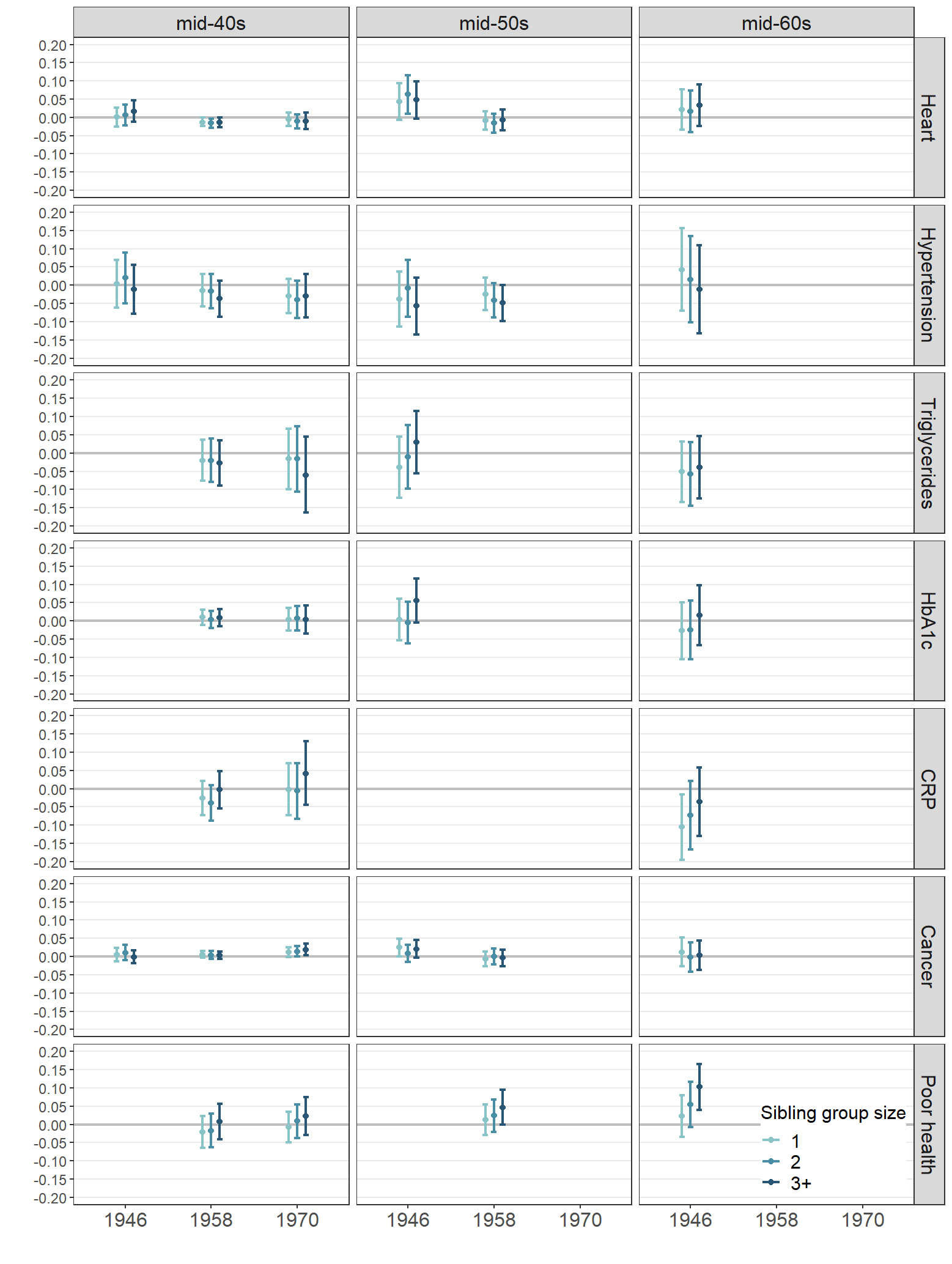
 Notes: Model specification 2 shown; regressions run separately for each outcome, age and cohort on complete cases. Outcomes are self-reported heart problems, high blood pressure (self-reported at age 55 only), high triglycerides, high Glycated haemoglobin (HbA1c), and high C-reactive protein (CRP), self-reported cancer diagnosis and self-assessed general health as fair/poor/very poor. Models adjust for cohort members’ (CM) sex and birth order, maternal age at CM’s birth, maternal education, whether the CM was breastfed, paternal social class and parental separation by age 10/11.*

**Supplementary Table S6 Summary of regression results with interaction: Coefficients for sibship size groups (reference category: Only Child), parental social class and interaction of sibship size and social class, for each health outcome, separately by cohort and age**

| **1946 Age 43** | **Heart** | | |  |  | **Hypertension** | | | | **Cancer** | | | |
| --- | --- | --- | --- | --- | --- | --- | --- | --- | --- | --- | --- | --- | --- |
|  | **Coeff.** | **se** | **p-value** | | **CI** | **Coeff.** | **se** | **p-value** | **CI** | **Coeff.** | **se** | **p-value** | **CI** |
| Sibship size (Ref OC): 1 sibling | 0.034 | 0.040 | 0.406 | | -0.046 - 0.113 | 0.073 | 0.092 | 0.431 | -0.108 - 0.253 | 0.022 | 0.018 | 0.224 | -0.013 - 0.056 |
| 2 siblings | 0.013 | 0.039 | 0.728 | | -0.062 - 0.089 | 0.153 | 0.097 | 0.117 | -0.038 - 0.343 | 0.008 | 0.015 | 0.579 | -0.021 - 0.037 |
| 3+ siblings | -0.006 | 0.038 | 0.870 | | -0.081 - 0.069 | 0.086 | 0.101 | 0.392 | -0.111 - 0.283 | 0.001 | 0.016 | 0.936 | -0.031 - 0.033 |
| Social class (ref I): II | 0.012 | 0.037 | 0.749 | | -0.061 - 0.085 | 0.068 | 0.094 | 0.467 | -0.116 - 0.253 | 0.011 | 0.019 | 0.554 | -0.026 - 0.049 |
| III non-manual | 0.001 | 0.033 | 0.968 | | -0.064 - 0.067 | 0.140 | 0.087 | 0.109 | -0.031 - 0.311 | 0.010 | 0.013 | 0.444 | -0.016 - 0.036 |
| III manual | 0.016 | 0.036 | 0.656 | | -0.054 - 0.085 | 0.171 | 0.086 | 0.048 | 0.002 - 0.340 | 0.021 | 0.015 | 0.160 | -0.008 - 0.051 |
| IV | 0.036 | 0.044 | 0.423 | | -0.051 - 0.123 | 0.157 | 0.096 | 0.103 | -0.032 - 0.345 | 0.022 | 0.014 | 0.130 | -0.006 - 0.050 |
| V | 0.012 | 0.051 | 0.816 | | -0.089 - 0.112 | 0.065 | 0.124 | 0.602 | -0.179 - 0.308 | 0.022 | 0.040 | 0.576 | -0.056 - 0.101 |
| n/a, unemp. | 0.046 | 0.055 | 0.402 | | -0.062 - 0.154 | 0.163 | 0.116 | 0.160 | -0.064 - 0.390 | 0.056 | 0.043 | 0.188 | -0.028 - 0.140 |
| Interactions: 1sib#II | -0.027 | 0.048 | 0.568 | | -0.121 - 0.067 | -0.002 | 0.120 | 0.987 | -0.237 - 0.233 | -0.013 | 0.026 | 0.611 | -0.064 - 0.037 |
| 1sib#III n-m | -0.007 | 0.044 | 0.868 | | -0.093 - 0.078 | -0.069 | 0.110 | 0.530 | -0.285 - 0.147 | -0.010 | 0.023 | 0.663 | -0.056 - 0.036 |
| 1sib#III m | -0.022 | 0.044 | 0.621 | | -0.109 - 0.065 | -0.085 | 0.103 | 0.412 | -0.287 - 0.118 | -0.015 | 0.024 | 0.523 | -0.063 - 0.032 |
| 1sib#IV | -0.045 | 0.052 | 0.387 | | -0.146 - 0.056 | -0.143 | 0.117 | 0.222 | -0.373 - 0.087 | -0.010 | 0.025 | 0.675 | -0.059 - 0.038 |
| 1sib#V | -0.005 | 0.066 | 0.944 | | -0.133 - 0.124 | 0.140 | 0.158 | 0.373 | -0.169 - 0.449 | 0.001 | 0.052 | 0.990 | -0.101 - 0.102 |
| 1sib#n/a | -0.059 | 0.068 | 0.386 | | -0.193 - 0.075 | -0.086 | 0.140 | 0.540 | -0.359 - 0.188 | -0.049 | 0.053 | 0.352 | -0.152 - 0.054 |
| 2sibs#II | 0.020 | 0.050 | 0.690 | | -0.078 - 0.119 | -0.135 | 0.123 | 0.276 | -0.376 - 0.107 | -0.005 | 0.027 | 0.844 | -0.058 - 0.047 |
| 2sibs#III n-m | -0.001 | 0.042 | 0.980 | | -0.083 - 0.081 | -0.176 | 0.117 | 0.133 | -0.405 - 0.053 | -0.003 | 0.022 | 0.881 | -0.046 - 0.040 |
| 2sibs#III m | -0.001 | 0.044 | 0.980 | | -0.087 - 0.085 | -0.149 | 0.114 | 0.189 | -0.372 - 0.074 | 0.000 | 0.022 | 0.996 | -0.043 - 0.043 |
| 2sibs#IV | -0.015 | 0.053 | 0.775 | | -0.118 - 0.088 | -0.157 | 0.121 | 0.197 | -0.394 - 0.081 | 0.018 | 0.026 | 0.482 | -0.033 - 0.070 |
| 2sibs#V | 0.015 | 0.065 | 0.813 | | -0.111 - 0.142 | -0.116 | 0.157 | 0.460 | -0.423 - 0.192 | -0.013 | 0.046 | 0.783 | -0.102 - 0.077 |
| 2sibs#n/a | -0.026 | 0.070 | 0.712 | | -0.162 - 0.111 | -0.202 | 0.143 | 0.157 | -0.483 - 0.078 | -0.039 | 0.055 | 0.476 | -0.146 - 0.068 |
| 3+sibs#II | 0.016 | 0.049 | 0.752 | | -0.081 - 0.112 | -0.047 | 0.124 | 0.703 | -0.291 - 0.196 | 0.009 | 0.026 | 0.718 | -0.041 - 0.059 |
| 3+sibs#III n-m | 0.012 | 0.043 | 0.779 | | -0.073 - 0.097 | -0.184 | 0.120 | 0.127 | -0.420 - 0.052 | 0.009 | 0.025 | 0.710 | -0.040 - 0.059 |
| 3+sibs#III m | 0.028 | 0.043 | 0.512 | | -0.056 - 0.112 | -0.147 | 0.112 | 0.190 | -0.366 - 0.073 | 0.001 | 0.022 | 0.970 | -0.043 - 0.045 |
| 3+sibs#IV | 0.002 | 0.051 | 0.969 | | -0.097 - 0.101 | -0.124 | 0.122 | 0.309 | -0.363 - 0.115 | -0.007 | 0.021 | 0.736 | -0.047 - 0.034 |
| 3+sibs#V | 0.057 | 0.060 | 0.343 | | -0.061 - 0.175 | -0.046 | 0.147 | 0.753 | -0.334 - 0.242 | 0.011 | 0.046 | 0.813 | -0.079 - 0.101 |
| 3sibs#n/a | 0.021 | 0.069 | 0.763 | | -0.114 - 0.155 | -0.064 | 0.150 | 0.672 | -0.357 - 0.230 | -0.038 | 0.049 | 0.437 | -0.134 - 0.058 |

| **1958 Age 44/46** | **Heart** |  |  |  | **Hypertension** | | | |
| --- | --- | --- | --- | --- | --- | --- | --- | --- |
|  | **Coeff.** | **se** | **p-value** | **CI** | **Coeff.** | **se** | **p-value** | **CI** |
| Sibship size (Ref OC): 1 sibling | -0.004 | 0.027 | 0.884 | -0.057 - 0.049 | -0.059 | 0.110 | 0.589 | -0.275 - 0.156 |
| 2 siblings | 0.010 | 0.028 | 0.724 | -0.045 - 0.064 | -0.058 | 0.113 | 0.607 | -0.280 - 0.164 |
| 3+ siblings | -0.004 | 0.028 | 0.885 | -0.059 - 0.051 | -0.090 | 0.114 | 0.432 | -0.313 - 0.134 |
| Social class (ref I): II | 0.018 | 0.028 | 0.520 | -0.037 - 0.074 | -0.008 | 0.116 | 0.945 | -0.236 - 0.220 |
| III non-manual | 0.029 | 0.030 | 0.348 | -0.031 - 0.088 | 0.046 | 0.124 | 0.709 | -0.196 - 0.289 |
| III manual | 0.021 | 0.026 | 0.424 | -0.030 - 0.071 | -0.004 | 0.106 | 0.972 | -0.211 - 0.204 |
| IV | 0.028 | 0.030 | 0.351 | -0.031 - 0.088 | -0.031 | 0.121 | 0.800 | -0.268 - 0.207 |
| V | 0.001 | 0.034 | 0.986 | -0.065 - 0.066 | 0.049 | 0.136 | 0.720 | -0.218 - 0.315 |
| Interactions: 1sib#II | -0.003 | 0.031 | 0.918 | -0.064 - 0.058 | 0.058 | 0.126 | 0.645 | -0.189 - 0.306 |
| 1sib#III n-m | -0.005 | 0.033 | 0.879 | -0.070 - 0.060 | -0.020 | 0.134 | 0.882 | -0.282 - 0.243 |
| 1sib#III m | -0.016 | 0.028 | 0.579 | -0.071 - 0.040 | 0.063 | 0.115 | 0.584 | -0.162 - 0.288 |
| 1sib#IV | -0.000 | 0.034 | 0.998 | -0.066 - 0.066 | 0.127 | 0.133 | 0.339 | -0.133 - 0.387 |
| 1sib#V | 0.028 | 0.037 | 0.455 | -0.045 - 0.101 | 0.117 | 0.151 | 0.439 | -0.179 - 0.412 |
| 2sibs#II | -0.024 | 0.032 | 0.458 | -0.086 - 0.039 | 0.050 | 0.129 | 0.697 | -0.203 - 0.304 |
| 2sibs#III n-m | -0.031 | 0.034 | 0.367 | -0.097 - 0.036 | 0.016 | 0.137 | 0.909 | -0.253 - 0.284 |
| 2sibs#III m | -0.022 | 0.029 | 0.441 | -0.079 - 0.034 | 0.055 | 0.118 | 0.642 | -0.176 - 0.286 |
| 2sibs#IV | -0.036 | 0.034 | 0.289 | -0.103 - 0.031 | 0.089 | 0.135 | 0.510 | -0.176 - 0.354 |
| 2sibs#V | -0.002 | 0.038 | 0.952 | -0.076 - 0.072 | 0.056 | 0.152 | 0.714 | -0.243 - 0.354 |
| 3+sibs#II | -0.019 | 0.032 | 0.549 | -0.082 - 0.044 | 0.048 | 0.130 | 0.715 | -0.208 - 0.303 |
| 3+sibs#III n-m | -0.024 | 0.034 | 0.483 | -0.091 - 0.043 | -0.004 | 0.138 | 0.978 | -0.275 - 0.268 |
| 3+sibs#III m | -0.006 | 0.029 | 0.847 | -0.063 - 0.051 | 0.080 | 0.118 | 0.498 | -0.151 - 0.311 |
| 3+sibs#IV | -0.018 | 0.034 | 0.586 | -0.085 - 0.048 | 0.098 | 0.134 | 0.465 | -0.165 - 0.360 |
| 3+sibs#V | -0.005 | 0.037 | 0.900 | -0.077 - 0.068 | 0.035 | 0.148 | 0.812 | -0.255 - 0.326 |

| **1958 Age 44/46** | **High triglycerides** | | | | **High glycated haemoglobin** | | | | **High CRP** | | | |
| --- | --- | --- | --- | --- | --- | --- | --- | --- | --- | --- | --- | --- |
|  | **Coeff.** | **se** | **p-value** | **CI** | **Coeff.** | **se** | **p-value** | **CI** | **Coeff.** | **se** | **p-value** | **CI** |
| Sibship size (Ref OC): 1 sibling | 0.271 | 0.147 | 0.065 | -0.017 - 0.559 | 0.030 | 0.051 | 0.556 | -0.069 - 0.129 | 0.086 | 0.114 | 0.451 | -0.138 - 0.309 |
| 2 siblings | 0.111 | 0.151 | 0.461 | -0.185 - 0.407 | 0.040 | 0.052 | 0.437 | -0.061 - 0.142 | 0.156 | 0.117 | 0.181 | -0.073 - 0.386 |
| 3+ siblings | 0.188 | 0.151 | 0.213 | -0.108 - 0.484 | 0.071 | 0.052 | 0.173 | -0.031 - 0.172 | 0.041 | 0.117 | 0.725 | -0.189 - 0.271 |
| Social class (ref I): II | 0.266 | 0.154 | 0.085 | -0.036 - 0.568 | 0.049 | 0.053 | 0.356 | -0.055 - 0.154 | 0.116 | 0.120 | 0.338 | -0.121 - 0.352 |
| III non-manual | 0.357 | 0.163 | 0.029 | 0.037 - 0.676 | 0.030 | 0.056 | 0.590 | -0.080 - 0.141 | 0.126 | 0.126 | 0.321 | -0.122 - 0.374 |
| III manual | 0.246 | 0.142 | 0.082 | -0.032 - 0.525 | 0.020 | 0.049 | 0.679 | -0.075 - 0.116 | 0.102 | 0.110 | 0.351 | -0.113 - 0.317 |
| IV | 0.317 | 0.161 | 0.049 | 0.002 - 0.632 | 0.027 | 0.055 | 0.629 | -0.081 - 0.135 | 0.028 | 0.125 | 0.824 | -0.217 - 0.272 |
| V | 0.521 | 0.181 | 0.004 | 0.166 - 0.875 | 0.110 | 0.062 | 0.078 | -0.012 - 0.231 | 0.231 | 0.142 | 0.103 | -0.047 - 0.508 |
| Interactions: 1sib#II | -0.325 | 0.166 | 0.050 | -0.651 - 0.001 | -0.052 | 0.058 | 0.372 | -0.165 - 0.062 | -0.131 | 0.131 | 0.316 | -0.387 - 0.125 |
| 1sib#III n-m | -0.379 | 0.175 | 0.031 | -0.722 - -0.035 | -0.038 | 0.061 | 0.531 | -0.157 - 0.081 | -0.140 | 0.137 | 0.306 | -0.408 - 0.128 |
| 1sib#III m | -0.261 | 0.152 | 0.086 | -0.560 - 0.037 | -0.009 | 0.053 | 0.859 | -0.112 - 0.094 | -0.082 | 0.119 | 0.491 | -0.314 - 0.151 |
| 1sib#IV | -0.345 | 0.175 | 0.048 | -0.687 - -0.003 | -0.037 | 0.060 | 0.539 | -0.155 - 0.081 | 0.014 | 0.136 | 0.917 | -0.253 - 0.281 |
| 1sib#V | -0.518 | 0.198 | 0.009 | -0.907 - -0.129 | -0.147 | 0.069 | 0.033 | -0.281 - -0.012 | -0.248 | 0.157 | 0.113 | -0.555 - 0.059 |
| 2sibs#II | -0.221 | 0.170 | 0.194 | -0.553 - 0.112 | -0.072 | 0.059 | 0.222 | -0.188 - 0.044 | -0.210 | 0.133 | 0.115 | -0.471 - 0.051 |
| 2sibs#III n-m | -0.223 | 0.179 | 0.214 | -0.574 - 0.129 | -0.038 | 0.062 | 0.544 | -0.159 - 0.084 | -0.217 | 0.140 | 0.120 | -0.491 - 0.057 |
| 2sibs#III m | -0.115 | 0.156 | 0.462 | -0.421 - 0.191 | -0.049 | 0.054 | 0.359 | -0.155 - 0.056 | -0.164 | 0.121 | 0.176 | -0.402 - 0.074 |
| 2sibs#IV | -0.111 | 0.178 | 0.534 | -0.459 - 0.238 | -0.024 | 0.061 | 0.698 | -0.144 - 0.096 | -0.094 | 0.139 | 0.496 | -0.366 - 0.177 |
| 2sibs#V | -0.361 | 0.200 | 0.071 | -0.753 - 0.031 | -0.096 | 0.069 | 0.164 | -0.231 - 0.039 | -0.341 | 0.157 | 0.030 | -0.650 - -0.033 |
| 3+sibs#II | -0.281 | 0.170 | 0.099 | -0.615 - 0.053 | -0.107 | 0.059 | 0.071 | -0.223 - 0.009 | -0.136 | 0.134 | 0.310 | -0.398 - 0.127 |
| 3+sibs#III n-m | -0.297 | 0.180 | 0.099 | -0.650 - 0.056 | -0.097 | 0.062 | 0.120 | -0.219 - 0.025 | -0.072 | 0.141 | 0.609 | -0.348 - 0.204 |
| 3+sibs#III m | -0.184 | 0.155 | 0.237 | -0.489 - 0.121 | -0.069 | 0.054 | 0.196 | -0.174 - 0.036 | -0.007 | 0.121 | 0.952 | -0.245 - 0.230 |
| 3+sibs#IV | -0.224 | 0.175 | 0.201 | -0.567 - 0.119 | -0.072 | 0.060 | 0.234 | -0.189 - 0.046 | 0.015 | 0.137 | 0.911 | -0.253 - 0.283 |
| 3+sibs#V | -0.439 | 0.195 | 0.024 | -0.820 - -0.058 | -0.155 | 0.067 | 0.021 | -0.287 - -0.024 | -0.180 | 0.153 | 0.241 | -0.480 - 0.121 |

| **1958 Age 44/46** | **Cancer** |  |  |  | **General Health** | | |  |  |
| --- | --- | --- | --- | --- | --- | --- | --- | --- | --- |
|  | **Coeff.** | **se** | **p-value** | **CI** | **Coeff.** | **se** | **p-value** | | **CI** |
| Sibship size (Ref OC): 1 sibling | -0.001 | 0.023 | 0.959 | -0.046 - 0.043 | 0.015 | 0.098 | 0.877 | | -0.177 - 0.207 |
| 2 siblings | -0.001 | 0.023 | 0.964 | -0.047 - 0.045 | -0.045 | 0.101 | 0.656 | | -0.242 - 0.152 |
| 3+ siblings | -0.002 | 0.024 | 0.938 | -0.048 - 0.044 | 0.069 | 0.102 | 0.499 | | -0.130 - 0.268 |
| Social class (ref I): II | -0.001 | 0.024 | 0.952 | -0.048 - 0.045 | 0.125 | 0.103 | 0.224 | | -0.077 - 0.327 |
| III non-manual | -0.000 | 0.026 | 0.996 | -0.050 - 0.050 | 0.102 | 0.110 | 0.357 | | -0.115 - 0.318 |
| III manual | 0.003 | 0.022 | 0.904 | -0.040 - 0.045 | 0.044 | 0.094 | 0.636 | | -0.139 - 0.228 |
| IV | -0.002 | 0.026 | 0.946 | -0.052 - 0.049 | 0.009 | 0.110 | 0.933 | | -0.206 - 0.225 |
| V | 0.042 | 0.028 | 0.133 | -0.013 - 0.098 | 0.131 | 0.121 | 0.282 | | -0.108 - 0.369 |
| Interactions: 1sib#II | 0.015 | 0.026 | 0.571 | -0.037 - 0.066 | -0.074 | 0.113 | 0.512 | | -0.295 - 0.147 |
| 1sib#III n-m | 0.022 | 0.028 | 0.428 | -0.033 - 0.077 | -0.081 | 0.120 | 0.500 | | -0.317 - 0.154 |
| 1sib#III m | 0.003 | 0.024 | 0.908 | -0.044 - 0.049 | -0.006 | 0.102 | 0.952 | | -0.207 - 0.195 |
| 1sib#IV | 0.008 | 0.028 | 0.763 | -0.047 - 0.064 | 0.064 | 0.121 | 0.596 | | -0.173 - 0.302 |
| 1sib#V | -0.017 | 0.031 | 0.579 | -0.079 - 0.044 | -0.030 | 0.135 | 0.827 | | -0.295 - 0.235 |
| 2sibs#II | 0.009 | 0.027 | 0.731 | -0.043 - 0.062 | -0.071 | 0.116 | 0.541 | | -0.297 - 0.156 |
| 2sibs#III n-m | 0.011 | 0.029 | 0.699 | -0.045 - 0.067 | 0.018 | 0.123 | 0.883 | | -0.223 - 0.259 |
| 2sibs#III m | 0.002 | 0.024 | 0.950 | -0.046 - 0.049 | 0.026 | 0.105 | 0.801 | | -0.180 - 0.232 |
| 2sibs#IV | 0.015 | 0.029 | 0.605 | -0.041 - 0.071 | 0.156 | 0.123 | 0.206 | | -0.086 - 0.397 |
| 2sibs#V | -0.016 | 0.032 | 0.608 | -0.078 - 0.046 | 0.067 | 0.137 | 0.623 | | -0.201 - 0.335 |
| 3+sibs#II | 0.009 | 0.027 | 0.750 | -0.044 - 0.062 | -0.207 | 0.117 | 0.077 | | -0.436 - 0.022 |
| 3+sibs#III n-m | 0.010 | 0.029 | 0.719 | -0.046 - 0.067 | -0.121 | 0.125 | 0.332 | | -0.365 - 0.124 |
| 3+sibs#III m | 0.005 | 0.024 | 0.843 | -0.043 - 0.053 | -0.060 | 0.105 | 0.566 | | -0.267 - 0.146 |
| 3+sibs#IV | 0.011 | 0.028 | 0.705 | -0.045 - 0.066 | -0.060 | 0.122 | 0.621 | | -0.300 - 0.179 |
| 3+sibs#V | -0.035 | 0.031 | 0.257 | -0.096 - 0.026 | -0.151 | 0.133 | 0.259 | | -0.412 - 0.111 |

| **1970 Age 46** | **Heart** |  |  |  | **Hypertension** | | | |
| --- | --- | --- | --- | --- | --- | --- | --- | --- |
|  | **Coeff.** | **se** | **p-value** | **CI** | **Coeff.** | **se** | **p-value** | **CI** |
| Sibship size (Ref OC): 1 sibling | 0.040 | 0.042 | 0.342 | -0.042 - 0.122 | -0.032 | 0.111 | 0.771 | -0.250 - 0.185 |
| 2 siblings | 0.010 | 0.043 | 0.823 | -0.075 - 0.094 | -0.040 | 0.115 | 0.726 | -0.266 - 0.185 |
| 3+ siblings | 0.018 | 0.049 | 0.721 | -0.079 - 0.114 | 0.051 | 0.129 | 0.695 | -0.203 - 0.305 |
| Social class (ref I): II | 0.013 | 0.045 | 0.777 | -0.076 - 0.102 | 0.029 | 0.121 | 0.810 | -0.207 - 0.265 |
| III non-manual | 0.036 | 0.044 | 0.409 | -0.050 - 0.123 | 0.006 | 0.118 | 0.962 | -0.226 - 0.237 |
| III manual | 0.009 | 0.042 | 0.828 | -0.074 - 0.092 | 0.035 | 0.112 | 0.756 | -0.185 - 0.254 |
| IV | 0.013 | 0.047 | 0.779 | -0.078 - 0.104 | 0.035 | 0.124 | 0.775 | -0.207 - 0.278 |
| V | 0.064 | 0.060 | 0.282 | -0.053 - 0.182 | -0.093 | 0.159 | 0.560 | -0.405 - 0.220 |
| Interactions: 1sib#II | -0.034 | 0.047 | 0.470 | -0.127 - 0.059 | -0.018 | 0.127 | 0.886 | -0.266 - 0.230 |
| 1sib#III n-m | -0.053 | 0.046 | 0.256 | -0.143 - 0.038 | 0.024 | 0.124 | 0.849 | -0.219 - 0.266 |
| 1sib#III m | -0.035 | 0.044 | 0.429 | -0.121 - 0.051 | -0.015 | 0.117 | 0.898 | -0.244 - 0.214 |
| 1sib#IV | -0.022 | 0.049 | 0.651 | -0.118 - 0.074 | 0.025 | 0.130 | 0.846 | -0.230 - 0.280 |
| 1sib#V | -0.075 | 0.064 | 0.237 | -0.200 - 0.049 | 0.098 | 0.170 | 0.565 | -0.235 - 0.430 |
| 2sibs#II | -0.022 | 0.049 | 0.652 | -0.119 - 0.074 | -0.019 | 0.131 | 0.886 | -0.276 - 0.239 |
| 2sibs#III n-m | -0.023 | 0.048 | 0.639 | -0.117 - 0.072 | 0.077 | 0.129 | 0.552 | -0.176 - 0.329 |
| 2sibs#III m | -0.005 | 0.045 | 0.919 | -0.094 - 0.084 | -0.027 | 0.121 | 0.825 | -0.264 - 0.210 |
| 2sibs#IV | 0.005 | 0.050 | 0.914 | -0.093 - 0.104 | 0.010 | 0.134 | 0.939 | -0.252 - 0.273 |
| 2sibs#V | -0.087 | 0.065 | 0.181 | -0.214 - 0.040 | 0.071 | 0.173 | 0.680 | -0.268 - 0.411 |
| 3+sibs#II | -0.032 | 0.057 | 0.568 | -0.143 - 0.079 | -0.161 | 0.150 | 0.284 | -0.456 - 0.133 |
| 3+sibs#III n-m | -0.011 | 0.057 | 0.845 | -0.122 - 0.100 | -0.183 | 0.150 | 0.223 | -0.477 - 0.111 |
| 3+sibs#III m | -0.009 | 0.051 | 0.854 | -0.110 - 0.091 | -0.094 | 0.135 | 0.488 | -0.359 - 0.171 |
| 3+sibs#IV | -0.040 | 0.056 | 0.475 | -0.151 - 0.070 | -0.093 | 0.149 | 0.532 | -0.384 - 0.199 |
| 3+sibs#V | -0.082 | 0.070 | 0.238 | -0.219 - 0.054 | 0.094 | 0.185 | 0.611 | -0.269 - 0.457 |

| **1970 Age 46** | **High triglycerides** | | |  |  | **High glycated haemoglobin** | | | | **High CRP** | | | |
| --- | --- | --- | --- | --- | --- | --- | --- | --- | --- | --- | --- | --- | --- |
|  | **Coeff.** | **se** | **p-value** | | **CI** | **Coeff.** | **se** | **p-value** | **CI** | **Coeff.** | **se** | **p-value** | **CI** |
| Sibship size (Ref OC): 1 sibling | -0.060 | 0.278 | 0.828 | | -0.605 - 0.484 | 0.015 | 0.083 | 0.854 | -0.148 - 0.179 | -0.346 | 0.234 | 0.139 | -0.804 - 0.112 |
| 2 siblings | -0.048 | 0.281 | 0.865 | | -0.600 - 0.504 | -0.002 | 0.085 | 0.986 | -0.169 - 0.165 | -0.273 | 0.237 | 0.249 | -0.737 - 0.191 |
| 3+ siblings | 0.135 | 0.292 | 0.643 | | -0.438 - 0.709 | 0.031 | 0.093 | 0.739 | -0.151 - 0.212 | -0.080 | 0.246 | 0.746 | -0.562 - 0.403 |
| Social class (ref I): II | -0.108 | 0.286 | 0.705 | | -0.670 - 0.453 | 0.040 | 0.089 | 0.651 | -0.134 - 0.214 | -0.170 | 0.241 | 0.482 | -0.642 - 0.303 |
| III non-manual | 0.104 | 0.287 | 0.716 | | -0.459 - 0.668 | 0.021 | 0.088 | 0.807 | -0.151 - 0.193 | -0.353 | 0.242 | 0.144 | -0.827 - 0.121 |
| III manual | -0.029 | 0.280 | 0.918 | | -0.578 - 0.520 | 0.014 | 0.084 | 0.871 | -0.151 - 0.179 | -0.230 | 0.236 | 0.329 | -0.692 - 0.232 |
| IV | 0.082 | 0.292 | 0.778 | | -0.490 - 0.655 | 0.067 | 0.090 | 0.454 | -0.109 - 0.244 | -0.080 | 0.246 | 0.744 | -0.562 - 0.401 |
| V | -0.066 | 0.361 | 0.855 | | -0.774 - 0.642 | 0.079 | 0.115 | 0.491 | -0.146 - 0.304 | -0.467 | 0.304 | 0.124 | -1.062 - 0.129 |
| Interactions: 1sib#II | 0.154 | 0.294 | 0.599 | | -0.421 - 0.730 | -0.023 | 0.092 | 0.806 | -0.203 - 0.158 | 0.308 | 0.247 | 0.213 | -0.177 - 0.792 |
| 1sib#III n-m | -0.039 | 0.294 | 0.894 | | -0.615 - 0.537 | -0.000 | 0.091 | 0.996 | -0.178 - 0.177 | 0.418 | 0.247 | 0.091 | -0.067 - 0.903 |
| 1sib#III m | 0.040 | 0.286 | 0.887 | | -0.520 - 0.601 | 0.010 | 0.087 | 0.905 | -0.160 - 0.180 | 0.340 | 0.240 | 0.157 | -0.131 - 0.812 |
| 1sib#IV | -0.035 | 0.300 | 0.908 | | -0.623 - 0.554 | -0.034 | 0.093 | 0.720 | -0.217 - 0.150 | 0.191 | 0.253 | 0.448 | -0.304 - 0.687 |
| 1sib#V | 0.035 | 0.373 | 0.925 | | -0.697 - 0.767 | -0.071 | 0.120 | 0.555 | -0.306 - 0.164 | 0.637 | 0.314 | 0.043 | 0.021 - 1.253 |
| 2sibs#II | 0.123 | 0.299 | 0.681 | | -0.464 - 0.710 | 0.012 | 0.094 | 0.897 | -0.173 - 0.197 | 0.246 | 0.252 | 0.329 | -0.248 - 0.740 |
| 2sibs#III n-m | -0.114 | 0.299 | 0.704 | | -0.701 - 0.473 | 0.004 | 0.093 | 0.962 | -0.178 - 0.187 | 0.325 | 0.252 | 0.197 | -0.169 - 0.819 |
| 2sibs#III m | 0.035 | 0.290 | 0.904 | | -0.533 - 0.603 | 0.020 | 0.089 | 0.823 | -0.154 - 0.193 | 0.220 | 0.244 | 0.367 | -0.258 - 0.698 |
| 2sibs#IV | -0.027 | 0.304 | 0.928 | | -0.623 - 0.568 | -0.031 | 0.095 | 0.742 | -0.218 - 0.156 | 0.124 | 0.255 | 0.626 | -0.377 - 0.625 |
| 2sibs#V | 0.137 | 0.378 | 0.718 | | -0.605 - 0.879 | -0.082 | 0.123 | 0.507 | -0.322 - 0.159 | 0.476 | 0.318 | 0.134 | -0.148 - 1.101 |
| 3+sibs#II | -0.288 | 0.320 | 0.370 | | -0.916 - 0.341 | -0.073 | 0.105 | 0.483 | -0.279 - 0.132 | 0.077 | 0.270 | 0.775 | -0.452 - 0.606 |
| 3+sibs#III n-m | -0.306 | 0.324 | 0.345 | | -0.941 - 0.329 | 0.016 | 0.105 | 0.882 | -0.190 - 0.221 | 0.235 | 0.272 | 0.389 | -0.300 - 0.769 |
| 3+sibs#III m | -0.235 | 0.301 | 0.435 | | -0.826 - 0.355 | -0.020 | 0.096 | 0.837 | -0.208 - 0.169 | 0.073 | 0.253 | 0.774 | -0.424 - 0.570 |
| 3+sibs#IV | -0.313 | 0.316 | 0.323 | | -0.933 - 0.308 | -0.122 | 0.103 | 0.237 | -0.325 - 0.081 | -0.130 | 0.266 | 0.624 | -0.653 - 0.392 |
| 3+sibs#V | -0.084 | 0.388 | 0.829 | | -0.845 - 0.678 | -0.084 | 0.128 | 0.513 | -0.334 - 0.167 | 0.341 | 0.327 | 0.297 | -0.300 - 0.981 |

| **1970 Age 46** | **Cancer** |  |  |  | **General Health** | | | |
| --- | --- | --- | --- | --- | --- | --- | --- | --- |
|  | **Coeff.** | **se** | **p-value** | **CI** | **Coeff.** | **se** | **p-value** | **CI** |
| Sibship size (Ref OC): 1 sibling | 0.017 | 0.032 | 0.589 | -0.045 - 0.079 | -0.010 | 0.096 | 0.917 | -0.198 - 0.178 |
| 2 siblings | 0.033 | 0.033 | 0.313 | -0.031 - 0.097 | -0.027 | 0.099 | 0.785 | -0.221 - 0.167 |
| 3+ siblings | 0.038 | 0.037 | 0.313 | -0.036 - 0.111 | 0.044 | 0.113 | 0.695 | -0.177 - 0.266 |
| Social class (ref I): II | -0.002 | 0.034 | 0.960 | -0.069 - 0.066 | -0.018 | 0.104 | 0.861 | -0.221 - 0.185 |
| III non-manual | 0.000 | 0.034 | 1.000 | -0.066 - 0.066 | 0.003 | 0.101 | 0.980 | -0.196 - 0.201 |
| III manual | 0.001 | 0.032 | 0.981 | -0.062 - 0.064 | -0.014 | 0.097 | 0.889 | -0.203 - 0.176 |
| IV | 0.001 | 0.035 | 0.983 | -0.068 - 0.070 | 0.000 | 0.107 | 0.999 | -0.209 - 0.209 |
| V | -0.001 | 0.045 | 0.986 | -0.090 - 0.088 | 0.077 | 0.137 | 0.574 | -0.192 - 0.346 |
| Interactions: 1sib#II | -0.012 | 0.036 | 0.737 | -0.083 - 0.059 | 0.032 | 0.109 | 0.767 | -0.181 - 0.246 |
| 1sib#III n-m | -0.002 | 0.035 | 0.948 | -0.071 - 0.067 | -0.025 | 0.106 | 0.813 | -0.233 - 0.183 |
| 1sib#III m | 0.006 | 0.033 | 0.858 | -0.060 - 0.071 | 0.021 | 0.101 | 0.834 | -0.177 - 0.219 |
| 1sib#IV | 0.005 | 0.037 | 0.883 | -0.067 - 0.078 | 0.024 | 0.112 | 0.828 | -0.195 - 0.244 |
| 1sib#V | -0.013 | 0.048 | 0.795 | -0.107 - 0.082 | -0.007 | 0.146 | 0.963 | -0.292 - 0.279 |
| 2sibs#II | -0.005 | 0.037 | 0.899 | -0.078 - 0.068 | 0.021 | 0.113 | 0.849 | -0.200 - 0.243 |
| 2sibs#III n-m | -0.001 | 0.037 | 0.976 | -0.073 - 0.071 | 0.012 | 0.110 | 0.911 | -0.204 - 0.229 |
| 2sibs#III m | -0.018 | 0.034 | 0.610 | -0.085 - 0.050 | 0.042 | 0.104 | 0.687 | -0.162 - 0.246 |
| 2sibs#IV | -0.029 | 0.038 | 0.448 | -0.104 - 0.046 | 0.036 | 0.115 | 0.757 | -0.190 - 0.261 |
| 2sibs#V | -0.027 | 0.049 | 0.576 | -0.124 - 0.069 | -0.031 | 0.149 | 0.835 | -0.322 - 0.260 |
| 3+sibs#II | -0.016 | 0.043 | 0.715 | -0.100 - 0.068 | -0.078 | 0.130 | 0.546 | -0.333 - 0.176 |
| 3+sibs#III n-m | -0.014 | 0.043 | 0.743 | -0.099 - 0.070 | -0.002 | 0.130 | 0.985 | -0.258 - 0.253 |
| 3+sibs#III m | -0.014 | 0.039 | 0.717 | -0.091 - 0.062 | -0.039 | 0.118 | 0.739 | -0.271 - 0.192 |
| 3+sibs#IV | -0.007 | 0.043 | 0.868 | -0.091 - 0.077 | -0.081 | 0.129 | 0.530 | -0.334 - 0.172 |
| 3+sibs#V | -0.011 | 0.053 | 0.837 | -0.114 - 0.093 | -0.156 | 0.160 | 0.327 | -0.469 - 0.156 |

| **1946 Age 53** | **Heart** |  |  |  | **Hypertension** | | | |
| --- | --- | --- | --- | --- | --- | --- | --- | --- |
|  | **Coeff.** | **se** | **p-value** | **CI** | **Coeff.** | **se** | **p-value** | **CI** |
| Sibship size (Ref OC): 1 sibling | -0.042 | 0.143 | 0.772 | -0.322 - 0.239 | 0.154 | 0.134 | 0.250 | -0.109 - 0.417 |
| 2 siblings | -0.070 | 0.145 | 0.630 | -0.355 - 0.215 | 0.041 | 0.144 | 0.778 | -0.242 - 0.323 |
| 3+ siblings | -0.073 | 0.155 | 0.637 | -0.376 - 0.230 | 0.008 | 0.149 | 0.957 | -0.285 - 0.301 |
| Social class (ref I): II | -0.115 | 0.134 | 0.393 | -0.378 - 0.148 | 0.027 | 0.141 | 0.848 | -0.249 - 0.303 |
| III non-manual | -0.056 | 0.133 | 0.673 | -0.318 - 0.205 | 0.095 | 0.131 | 0.467 | -0.161 - 0.352 |
| III manual | -0.069 | 0.131 | 0.598 | -0.327 - 0.188 | 0.197 | 0.133 | 0.140 | -0.064 - 0.458 |
| IV | -0.040 | 0.139 | 0.771 | -0.312 - 0.232 | 0.174 | 0.142 | 0.219 | -0.104 - 0.452 |
| V | -0.105 | 0.143 | 0.464 | -0.385 - 0.175 | 0.093 | 0.167 | 0.575 | -0.233 - 0.420 |
| n/a, unemp. | -0.006 | 0.146 | 0.965 | -0.292 - 0.279 | 0.172 | 0.154 | 0.264 | -0.130 - 0.475 |
| Interactions: 1sib#II | 0.104 | 0.150 | 0.489 | -0.190 - 0.398 | -0.162 | 0.154 | 0.295 | -0.464 - 0.141 |
| 1sib#III n-m | 0.062 | 0.146 | 0.672 | -0.225 - 0.348 | -0.135 | 0.147 | 0.359 | -0.424 - 0.154 |
| 1sib#III m | 0.085 | 0.145 | 0.558 | -0.200 - 0.370 | -0.215 | 0.149 | 0.148 | -0.507 - 0.076 |
| 1sib#IV | 0.034 | 0.154 | 0.827 | -0.269 - 0.336 | -0.265 | 0.157 | 0.092 | -0.573 - 0.043 |
| 1sib#V | 0.115 | 0.165 | 0.487 | -0.209 - 0.439 | -0.101 | 0.192 | 0.599 | -0.477 - 0.275 |
| 1sib#n/a | -0.022 | 0.165 | 0.893 | -0.346 - 0.301 | -0.267 | 0.176 | 0.129 | -0.613 - 0.078 |
| 2sibs#II | 0.180 | 0.151 | 0.233 | -0.116 - 0.476 | -0.061 | 0.168 | 0.718 | -0.389 - 0.268 |
| 2sibs#III n-m | 0.068 | 0.150 | 0.650 | -0.227 - 0.363 | -0.032 | 0.157 | 0.841 | -0.340 - 0.277 |
| 2sibs#III m | 0.119 | 0.147 | 0.418 | -0.169 - 0.407 | -0.093 | 0.157 | 0.553 | -0.401 - 0.215 |
| 2sibs#IV | 0.086 | 0.156 | 0.583 | -0.219 - 0.391 | -0.068 | 0.170 | 0.690 | -0.401 - 0.265 |
| 2sibs#V | 0.098 | 0.167 | 0.557 | -0.229 - 0.425 | -0.004 | 0.206 | 0.984 | -0.409 - 0.400 |
| 2sibs#n/a | 0.077 | 0.169 | 0.651 | -0.255 - 0.408 | -0.060 | 0.195 | 0.756 | -0.442 - 0.321 |
| 3+sibs#II | 0.132 | 0.162 | 0.412 | -0.184 - 0.449 | -0.015 | 0.174 | 0.930 | -0.356 - 0.325 |
| 3+sibs#III n-m | 0.081 | 0.162 | 0.617 | -0.236 - 0.398 | -0.007 | 0.168 | 0.966 | -0.336 - 0.322 |
| 3+sibs#III m | 0.108 | 0.155 | 0.484 | -0.195 - 0.411 | -0.113 | 0.164 | 0.489 | -0.434 - 0.207 |
| 3+sibs#IV | 0.069 | 0.163 | 0.670 | -0.250 - 0.389 | -0.090 | 0.171 | 0.599 | -0.426 - 0.246 |
| 3+sibs#V | 0.152 | 0.173 | 0.378 | -0.186 - 0.490 | -0.006 | 0.198 | 0.974 | -0.394 - 0.381 |
| 3sibs#n/a | 0.112 | 0.178 | 0.531 | -0.238 - 0.461 | -0.057 | 0.187 | 0.759 | -0.423 - 0.309 |

| **1946 Age 53** | **High triglycerides** | | |  |  | **High glycated haemoglobin** | | | | **Cancer** | | | |
| --- | --- | --- | --- | --- | --- | --- | --- | --- | --- | --- | --- | --- | --- |
|  | **Coeff.** | **se** | **p-value** | | **CI** | **Coeff.** | **se** | **p-value** | **CI** | **Coeff.** | **se** | **p-value** | **CI** |
| Sibship size (Ref OC): 1 sibling | 0.094 | 0.143 | 0.511 | | -0.187 - 0.376 | 0.049 | 0.073 | 0.497 | -0.093 - 0.192 | 0.033 | 0.047 | 0.484 | -0.059 - 0.125 |
| 2 siblings | 0.083 | 0.148 | 0.574 | | -0.207 - 0.373 | -0.002 | 0.076 | 0.978 | -0.152 - 0.147 | 0.001 | 0.046 | 0.975 | -0.089 - 0.092 |
| 3+ siblings | 0.040 | 0.161 | 0.806 | | -0.277 - 0.356 | 0.008 | 0.090 | 0.926 | -0.169 - 0.185 | 0.018 | 0.059 | 0.764 | -0.099 - 0.134 |
| Social class (ref I): II | 0.085 | 0.151 | 0.572 | | -0.210 - 0.381 | 0.036 | 0.083 | 0.659 | -0.125 - 0.198 | 0.003 | 0.043 | 0.935 | -0.080 - 0.087 |
| III non-manual | 0.074 | 0.138 | 0.591 | | -0.196 - 0.344 | 0.071 | 0.078 | 0.367 | -0.083 - 0.225 | 0.019 | 0.042 | 0.646 | -0.063 - 0.101 |
| III manual | 0.088 | 0.136 | 0.518 | | -0.178 - 0.354 | 0.038 | 0.075 | 0.614 | -0.109 - 0.185 | 0.010 | 0.042 | 0.815 | -0.072 - 0.091 |
| IV | 0.135 | 0.148 | 0.360 | | -0.155 - 0.425 | 0.064 | 0.082 | 0.430 | -0.096 - 0.224 | 0.038 | 0.050 | 0.451 | -0.061 - 0.136 |
| V | 0.081 | 0.174 | 0.643 | | -0.261 - 0.422 | 0.020 | 0.106 | 0.851 | -0.187 - 0.227 | -0.002 | 0.049 | 0.964 | -0.098 - 0.094 |
| n/a, unemp. | 0.146 | 0.168 | 0.386 | | -0.183 - 0.475 | 0.047 | 0.099 | 0.637 | -0.148 - 0.241 | 0.024 | 0.053 | 0.652 | -0.080 - 0.127 |
| Interactions: 1sib#II | -0.159 | 0.177 | 0.367 | | -0.506 - 0.187 | -0.082 | 0.094 | 0.385 | -0.266 - 0.103 | -0.021 | 0.051 | 0.676 | -0.122 - 0.079 |
| 1sib#III n-m | -0.098 | 0.158 | 0.534 | | -0.407 - 0.211 | -0.074 | 0.092 | 0.425 | -0.254 - 0.107 | -0.033 | 0.049 | 0.496 | -0.129 - 0.062 |
| 1sib#III m | -0.101 | 0.155 | 0.518 | | -0.405 - 0.204 | -0.051 | 0.086 | 0.553 | -0.221 - 0.118 | -0.010 | 0.049 | 0.833 | -0.107 - 0.086 |
| 1sib#IV | -0.139 | 0.166 | 0.401 | | -0.465 - 0.186 | -0.088 | 0.095 | 0.357 | -0.274 - 0.099 | -0.007 | 0.059 | 0.907 | -0.123 - 0.109 |
| 1sib#V | -0.185 | 0.208 | 0.374 | | -0.594 - 0.223 | -0.058 | 0.127 | 0.645 | -0.306 - 0.190 | -0.017 | 0.060 | 0.776 | -0.134 - 0.100 |
| 1sib#n/a | -0.195 | 0.192 | 0.312 | | -0.572 - 0.183 | -0.059 | 0.119 | 0.621 | -0.291 - 0.174 | -0.010 | 0.064 | 0.882 | -0.136 - 0.117 |
| 2sibs#II | -0.190 | 0.178 | 0.286 | | -0.540 - 0.159 | -0.003 | 0.099 | 0.979 | -0.196 - 0.191 | -0.007 | 0.049 | 0.885 | -0.102 - 0.088 |
| 2sibs#III n-m | -0.097 | 0.167 | 0.561 | | -0.424 - 0.230 | -0.065 | 0.096 | 0.496 | -0.254 - 0.123 | 0.014 | 0.051 | 0.789 | -0.086 - 0.114 |
| 2sibs#III m | -0.051 | 0.157 | 0.746 | | -0.360 - 0.257 | -0.018 | 0.093 | 0.842 | -0.200 - 0.163 | 0.011 | 0.048 | 0.816 | -0.083 - 0.106 |
| 2sibs#IV | -0.080 | 0.172 | 0.640 | | -0.417 - 0.256 | -0.029 | 0.098 | 0.771 | -0.221 - 0.163 | -0.018 | 0.059 | 0.758 | -0.133 - 0.097 |
| 2sibs#V | -0.128 | 0.212 | 0.546 | | -0.542 - 0.287 | 0.014 | 0.125 | 0.910 | -0.231 - 0.260 | 0.005 | 0.057 | 0.937 | -0.108 - 0.117 |
| 2sibs#n/a | -0.171 | 0.195 | 0.379 | | -0.553 - 0.211 | -0.000 | 0.121 | 0.999 | -0.237 - 0.237 | -0.020 | 0.062 | 0.742 | -0.141 - 0.100 |
| 3+sibs#II | 0.002 | 0.189 | 0.993 | | -0.369 - 0.372 | 0.008 | 0.107 | 0.943 | -0.203 - 0.218 | 0.025 | 0.067 | 0.704 | -0.106 - 0.157 |
| 3+sibs#III n-m | -0.046 | 0.172 | 0.790 | | -0.383 - 0.291 | -0.049 | 0.110 | 0.657 | -0.264 - 0.166 | -0.016 | 0.064 | 0.798 | -0.142 - 0.109 |
| 3+sibs#III m | -0.013 | 0.169 | 0.936 | | -0.345 - 0.318 | 0.021 | 0.102 | 0.840 | -0.179 - 0.220 | -0.001 | 0.062 | 0.982 | -0.122 - 0.119 |
| 3+sibs#IV | -0.036 | 0.187 | 0.848 | | -0.402 - 0.331 | -0.004 | 0.113 | 0.972 | -0.226 - 0.218 | -0.014 | 0.068 | 0.836 | -0.148 - 0.119 |
| 3+sibs#V | -0.022 | 0.206 | 0.914 | | -0.426 - 0.382 | 0.021 | 0.129 | 0.872 | -0.233 - 0.275 | 0.013 | 0.068 | 0.843 | -0.120 - 0.147 |
| 3sibs#n/a | -0.052 | 0.208 | 0.802 | | -0.460 - 0.355 | 0.020 | 0.135 | 0.881 | -0.244 - 0.284 | -0.015 | 0.073 | 0.835 | -0.159 - 0.128 |

| **1958 Age 55** | **Heart** |  |  |  | **Hypertension** | | |  |
| --- | --- | --- | --- | --- | --- | --- | --- | --- |
|  | **Coeff.** | **se** | **p-value** | **CI** | **Coeff.** | **se** | **p-value** | **CI** |
| Sibship size (Ref OC): 1 sibling | -0.102 | 0.056 | 0.069 | -0.211 - 0.008 | -0.080 | 0.106 | 0.450 | -0.287 - 0.127 |
| 2 siblings | -0.055 | 0.058 | 0.339 | -0.168 - 0.058 | -0.063 | 0.110 | 0.566 | -0.278 - 0.152 |
| 3+ siblings | -0.038 | 0.058 | 0.517 | -0.152 - 0.076 | -0.074 | 0.110 | 0.505 | -0.290 - 0.143 |
| Social class (ref I): II | -0.089 | 0.059 | 0.131 | -0.205 - 0.026 | -0.097 | 0.112 | 0.387 | -0.316 - 0.122 |
| III non-manual | -0.089 | 0.062 | 0.146 | -0.210 - 0.031 | -0.073 | 0.117 | 0.530 | -0.302 - 0.155 |
| III manual | -0.049 | 0.054 | 0.360 | -0.155 - 0.056 | -0.023 | 0.102 | 0.822 | -0.223 - 0.177 |
| IV | -0.084 | 0.063 | 0.179 | -0.207 - 0.039 | 0.131 | 0.118 | 0.267 | -0.101 - 0.363 |
| V | -0.058 | 0.071 | 0.415 | -0.198 - 0.082 | -0.140 | 0.135 | 0.300 | -0.406 - 0.125 |
| Interactions: 1sib#II | 0.122 | 0.064 | 0.057 | -0.004 - 0.248 | 0.095 | 0.122 | 0.433 | -0.143 - 0.334 |
| 1sib#III n-m | 0.116 | 0.067 | 0.083 | -0.015 - 0.247 | 0.071 | 0.127 | 0.578 | -0.178 - 0.319 |
| 1sib#III m | 0.084 | 0.058 | 0.150 | -0.030 - 0.199 | 0.048 | 0.111 | 0.665 | -0.169 - 0.265 |
| 1sib#IV | 0.125 | 0.069 | 0.068 | -0.009 - 0.260 | -0.048 | 0.130 | 0.713 | -0.302 - 0.207 |
| 1sib#V | 0.072 | 0.078 | 0.357 | -0.082 - 0.226 | 0.140 | 0.149 | 0.345 | -0.151 - 0.432 |
| 2sibs#II | 0.050 | 0.066 | 0.453 | -0.080 - 0.179 | 0.078 | 0.125 | 0.535 | -0.168 - 0.323 |
| 2sibs#III n-m | 0.088 | 0.069 | 0.203 | -0.047 - 0.222 | 0.047 | 0.130 | 0.721 | -0.209 - 0.302 |
| 2sibs#III m | 0.025 | 0.060 | 0.677 | -0.093 - 0.143 | 0.021 | 0.114 | 0.851 | -0.203 - 0.246 |
| 2sibs#IV | 0.077 | 0.070 | 0.273 | -0.061 - 0.215 | -0.143 | 0.133 | 0.280 | -0.404 - 0.117 |
| 2sibs#V | 0.071 | 0.081 | 0.382 | -0.087 - 0.228 | 0.172 | 0.153 | 0.261 | -0.128 - 0.471 |
| 3+sibs#II | 0.048 | 0.067 | 0.471 | -0.083 - 0.179 | 0.061 | 0.126 | 0.631 | -0.187 - 0.308 |
| 3+sibs#III n-m | 0.077 | 0.070 | 0.268 | -0.059 - 0.214 | 0.050 | 0.132 | 0.707 | -0.209 - 0.309 |
| 3+sibs#III m | 0.020 | 0.060 | 0.740 | -0.098 - 0.138 | 0.024 | 0.114 | 0.836 | -0.201 - 0.248 |
| 3+sibs#IV | 0.071 | 0.069 | 0.306 | -0.065 - 0.207 | -0.114 | 0.131 | 0.383 | -0.372 - 0.143 |
| 3+sibs#V | 0.052 | 0.078 | 0.501 | -0.100 - 0.205 | 0.144 | 0.148 | 0.330 | -0.146 - 0.433 |

| **1958 Age 55** | **Cancer** |  |  |  | **General Health** | | |  |  |
| --- | --- | --- | --- | --- | --- | --- | --- | --- | --- |
|  | **Coeff.** | **se** | **p-value** | **CI** | **Coeff.** | **se** | **p-value** | **CI** | |
| Sibship size (Ref OC): 1 sibling | 0.027 | 0.047 | 0.571 | -0.065 - 0.118 | 0.061 | 0.094 | 0.513 | -0.122 - 0.245 | |
| 2 siblings | 0.009 | 0.048 | 0.860 | -0.087 - 0.104 | 0.017 | 0.097 | 0.862 | -0.173 - 0.207 | |
| 3+ siblings | 0.007 | 0.049 | 0.892 | -0.089 - 0.102 | 0.082 | 0.098 | 0.398 | -0.109 - 0.274 | |
| Social class (ref I): II | 0.012 | 0.049 | 0.807 | -0.085 - 0.109 | 0.078 | 0.099 | 0.427 | -0.115 - 0.272 | |
| III non-manual | 0.072 | 0.052 | 0.163 | -0.029 - 0.173 | 0.083 | 0.103 | 0.420 | -0.119 - 0.285 | |
| III manual | 0.032 | 0.045 | 0.473 | -0.056 - 0.121 | 0.047 | 0.090 | 0.602 | -0.130 - 0.224 | |
| IV | -0.006 | 0.052 | 0.906 | -0.109 - 0.096 | -0.005 | 0.105 | 0.962 | -0.210 - 0.200 | |
| V | 0.049 | 0.060 | 0.413 | -0.068 - 0.166 | 0.151 | 0.120 | 0.206 | -0.083 - 0.386 | |
| Interactions: 1sib#II | -0.023 | 0.054 | 0.669 | -0.129 - 0.083 | -0.046 | 0.108 | 0.672 | -0.257 - 0.166 | |
| 1sib#III n-m | -0.079 | 0.056 | 0.160 | -0.189 - 0.031 | -0.093 | 0.112 | 0.408 | -0.313 - 0.127 | |
| 1sib#III m | -0.039 | 0.049 | 0.424 | -0.136 - 0.057 | -0.047 | 0.098 | 0.635 | -0.239 - 0.146 | |
| 1sib#IV | 0.007 | 0.057 | 0.904 | -0.106 - 0.120 | -0.010 | 0.115 | 0.931 | -0.235 - 0.215 | |
| 1sib#V | -0.047 | 0.066 | 0.475 | -0.176 - 0.082 | -0.115 | 0.132 | 0.382 | -0.373 - 0.143 | |
| 2sibs#II | 0.015 | 0.055 | 0.790 | -0.094 - 0.123 | -0.059 | 0.111 | 0.592 | -0.277 - 0.158 | |
| 2sibs#III n-m | -0.053 | 0.058 | 0.357 | -0.166 - 0.060 | -0.035 | 0.115 | 0.763 | -0.261 - 0.191 | |
| 2sibs#III m | -0.004 | 0.051 | 0.943 | -0.103 - 0.096 | 0.008 | 0.101 | 0.939 | -0.190 - 0.206 | |
| 2sibs#IV | 0.011 | 0.059 | 0.845 | -0.104 - 0.127 | 0.074 | 0.117 | 0.530 | -0.156 - 0.304 | |
| 2sibs#V | -0.033 | 0.068 | 0.631 | -0.165 - 0.100 | -0.046 | 0.135 | 0.732 | -0.311 - 0.219 | |
| 3+sibs#II | 0.013 | 0.056 | 0.821 | -0.097 - 0.122 | -0.114 | 0.112 | 0.308 | -0.333 - 0.105 | |
| 3+sibs#III n-m | -0.031 | 0.058 | 0.601 | -0.145 - 0.084 | -0.087 | 0.117 | 0.455 | -0.316 - 0.142 | |
| 3+sibs#III m | -0.013 | 0.051 | 0.802 | -0.112 - 0.087 | -0.050 | 0.101 | 0.622 | -0.248 - 0.149 | |
| 3+sibs#IV | 0.029 | 0.058 | 0.615 | -0.085 - 0.143 | -0.014 | 0.116 | 0.905 | -0.241 - 0.214 | |
| 3+sibs#V | -0.044 | 0.065 | 0.501 | -0.172 - 0.084 | -0.146 | 0.131 | 0.265 | -0.402 - 0.110 | |

| **1946 Age 63** | **Heart** |  |  |  | **Hypertension** | | | |
| --- | --- | --- | --- | --- | --- | --- | --- | --- |
|  | **Coeff.** | **se** | **p-value** | **CI** | **Coeff.** | **se** | **p-value** | **CI** |
| Sibship size (Ref OC): 1 sibling | 0.028 | 0.089 | 0.756 | -0.146 - 0.202 | 0.000 | 0.145 | 0.998 | -0.283 - 0.284 |
| 2 siblings | 0.000 | 0.090 | 0.997 | -0.176 - 0.176 | 0.019 | 0.163 | 0.908 | -0.301 - 0.339 |
| 3+ siblings | 0.045 | 0.108 | 0.680 | -0.168 - 0.257 | 0.055 | 0.175 | 0.753 | -0.288 - 0.399 |
| Social class (ref I): II | 0.001 | 0.093 | 0.992 | -0.181 - 0.183 | -0.031 | 0.145 | 0.830 | -0.315 - 0.253 |
| III non-manual | 0.039 | 0.090 | 0.664 | -0.137 - 0.214 | 0.030 | 0.141 | 0.833 | -0.246 - 0.305 |
| III manual | 0.036 | 0.091 | 0.689 | -0.142 - 0.214 | 0.042 | 0.137 | 0.758 | -0.226 - 0.310 |
| IV | 0.025 | 0.098 | 0.800 | -0.168 - 0.217 | 0.026 | 0.156 | 0.869 | -0.280 - 0.332 |
| V | -0.045 | 0.103 | 0.664 | -0.247 - 0.157 | -0.049 | 0.169 | 0.773 | -0.379 - 0.282 |
| n/a, unemp. | 0.054 | 0.102 | 0.595 | -0.146 - 0.255 | 0.072 | 0.162 | 0.658 | -0.245 - 0.388 |
| Interactions: 1sib#II | 0.020 | 0.105 | 0.851 | -0.185 - 0.225 | 0.017 | 0.165 | 0.918 | -0.306 - 0.340 |
| 1sib#III n-m | 0.005 | 0.101 | 0.964 | -0.194 - 0.203 | -0.003 | 0.151 | 0.983 | -0.299 - 0.293 |
| 1sib#III m | -0.010 | 0.101 | 0.919 | -0.208 - 0.187 | -0.001 | 0.149 | 0.992 | -0.294 - 0.291 |
| 1sib#IV | 0.004 | 0.111 | 0.968 | -0.212 - 0.221 | -0.012 | 0.167 | 0.942 | -0.339 - 0.315 |
| 1sib#V | 0.031 | 0.124 | 0.803 | -0.211 - 0.273 | 0.126 | 0.191 | 0.510 | -0.249 - 0.501 |
| 1sib#n/a | -0.078 | 0.122 | 0.525 | -0.318 - 0.162 | -0.046 | 0.187 | 0.806 | -0.413 - 0.321 |
| 2sibs#II | 0.021 | 0.107 | 0.842 | -0.189 - 0.231 | 0.045 | 0.177 | 0.797 | -0.302 - 0.392 |
| 2sibs#III n-m | -0.037 | 0.100 | 0.713 | -0.234 - 0.160 | -0.054 | 0.169 | 0.750 | -0.385 - 0.277 |
| 2sibs#III m | 0.013 | 0.102 | 0.899 | -0.187 - 0.213 | -0.025 | 0.162 | 0.876 | -0.342 - 0.291 |
| 2sibs#IV | 0.004 | 0.110 | 0.972 | -0.212 - 0.220 | -0.004 | 0.190 | 0.982 | -0.378 - 0.369 |
| 2sibs#V | 0.053 | 0.120 | 0.660 | -0.183 - 0.288 | -0.004 | 0.199 | 0.983 | -0.394 - 0.385 |
| 2sibs#n/a | -0.067 | 0.122 | 0.579 | -0.306 - 0.171 | -0.081 | 0.196 | 0.678 | -0.465 - 0.302 |
| 3+sibs#II | -0.000 | 0.125 | 0.997 | -0.246 - 0.245 | -0.018 | 0.192 | 0.927 | -0.395 - 0.360 |
| 3+sibs#III n-m | -0.017 | 0.127 | 0.895 | -0.265 - 0.231 | -0.047 | 0.188 | 0.801 | -0.415 - 0.321 |
| 3+sibs#III m | -0.049 | 0.117 | 0.677 | -0.279 - 0.181 | -0.091 | 0.184 | 0.620 | -0.451 - 0.269 |
| 3+sibs#IV | -0.028 | 0.127 | 0.825 | -0.276 - 0.220 | -0.058 | 0.196 | 0.768 | -0.442 - 0.326 |
| 3+sibs#V | 0.047 | 0.132 | 0.722 | -0.212 - 0.306 | 0.025 | 0.209 | 0.903 | -0.384 - 0.435 |
| 3sibs#n/a | -0.024 | 0.140 | 0.862 | -0.298 - 0.249 | -0.072 | 0.212 | 0.733 | -0.489 - 0.344 |

| **1946 Age 63** | **High triglycerides** | | |  |  | **High glycated haemoglobin** | | | | **High CRP** | | | |
| --- | --- | --- | --- | --- | --- | --- | --- | --- | --- | --- | --- | --- | --- |
|  | **Coeff.** | **se** | **p-value** | | **CI** | **Coeff.** | **se** | **p-value** | **CI** | **Coeff.** | **se** | **p-value** | **CI** |
| Sibship size (Ref OC): 1 sibling | 0.017 | 0.119 | 0.887 | | -0.216 - 0.250 | -0.078 | 0.106 | 0.464 | -0.285 - 0.130 | 0.092 | 0.119 | 0.436 | -0.140 - 0.325 |
| 2 siblings | -0.040 | 0.124 | 0.746 | | -0.284 - 0.203 | -0.077 | 0.109 | 0.478 | -0.290 - 0.136 | 0.009 | 0.127 | 0.942 | -0.240 - 0.259 |
| 3+ siblings | -0.071 | 0.135 | 0.599 | | -0.335 - 0.194 | -0.019 | 0.130 | 0.881 | -0.274 - 0.235 | 0.049 | 0.137 | 0.720 | -0.220 - 0.318 |
| Social class (ref I): II | -0.029 | 0.117 | 0.801 | | -0.258 - 0.199 | -0.037 | 0.115 | 0.750 | -0.263 - 0.189 | 0.070 | 0.127 | 0.579 | -0.178 - 0.319 |
| III non-manual | 0.009 | 0.112 | 0.938 | | -0.210 - 0.228 | 0.006 | 0.109 | 0.959 | -0.208 - 0.219 | 0.053 | 0.121 | 0.660 | -0.184 - 0.291 |
| III manual | 0.025 | 0.117 | 0.832 | | -0.205 - 0.255 | 0.001 | 0.107 | 0.993 | -0.209 - 0.211 | 0.106 | 0.114 | 0.349 | -0.116 - 0.329 |
| IV | 0.030 | 0.123 | 0.807 | | -0.211 - 0.272 | -0.020 | 0.114 | 0.859 | -0.243 - 0.203 | 0.085 | 0.127 | 0.504 | -0.163 - 0.333 |
| V | 0.122 | 0.155 | 0.431 | | -0.181 - 0.425 | 0.072 | 0.146 | 0.623 | -0.215 - 0.358 | 0.138 | 0.162 | 0.395 | -0.180 - 0.456 |
| n/a, unemp. | 0.031 | 0.137 | 0.819 | | -0.238 - 0.301 | 0.004 | 0.137 | 0.978 | -0.265 - 0.272 | 0.063 | 0.157 | 0.686 | -0.244 - 0.370 |
| Interactions: 1sib#II | -0.047 | 0.134 | 0.728 | | -0.310 - 0.216 | 0.028 | 0.127 | 0.829 | -0.222 - 0.277 | -0.178 | 0.147 | 0.225 | -0.466 - 0.110 |
| 1sib#III n-m | -0.062 | 0.127 | 0.625 | | -0.310 - 0.186 | 0.008 | 0.120 | 0.944 | -0.227 - 0.244 | -0.142 | 0.140 | 0.310 | -0.417 - 0.132 |
| 1sib#III m | -0.058 | 0.133 | 0.664 | | -0.319 - 0.203 | 0.057 | 0.120 | 0.634 | -0.178 - 0.292 | -0.151 | 0.132 | 0.255 | -0.410 - 0.109 |
| 1sib#IV | -0.102 | 0.140 | 0.466 | | -0.377 - 0.172 | 0.034 | 0.125 | 0.785 | -0.210 - 0.278 | -0.150 | 0.141 | 0.290 | -0.427 - 0.127 |
| 1sib#V | -0.173 | 0.196 | 0.378 | | -0.557 - 0.211 | -0.015 | 0.175 | 0.934 | -0.358 - 0.329 | -0.182 | 0.194 | 0.348 | -0.562 - 0.198 |
| 1sib#n/a | -0.128 | 0.158 | 0.420 | | -0.438 - 0.182 | 0.040 | 0.159 | 0.800 | -0.271 - 0.351 | -0.123 | 0.179 | 0.491 | -0.473 - 0.227 |
| 2sibs#II | -0.011 | 0.142 | 0.939 | | -0.290 - 0.268 | 0.069 | 0.133 | 0.603 | -0.192 - 0.331 | -0.045 | 0.161 | 0.780 | -0.361 - 0.271 |
| 2sibs#III n-m | -0.061 | 0.135 | 0.652 | | -0.325 - 0.204 | -0.017 | 0.125 | 0.892 | -0.262 - 0.228 | -0.012 | 0.151 | 0.936 | -0.308 - 0.284 |
| 2sibs#III m | -0.027 | 0.138 | 0.847 | | -0.297 - 0.244 | 0.028 | 0.122 | 0.817 | -0.210 - 0.267 | -0.075 | 0.145 | 0.605 | -0.360 - 0.209 |
| 2sibs#IV | -0.048 | 0.147 | 0.744 | | -0.337 - 0.241 | 0.113 | 0.131 | 0.387 | -0.143 - 0.370 | -0.077 | 0.151 | 0.608 | -0.373 - 0.219 |
| 2sibs#V | -0.157 | 0.187 | 0.402 | | -0.525 - 0.210 | -0.028 | 0.173 | 0.871 | -0.367 - 0.311 | -0.094 | 0.200 | 0.640 | -0.486 - 0.299 |
| 2sibs#n/a | -0.082 | 0.158 | 0.604 | | -0.391 - 0.227 | 0.099 | 0.156 | 0.527 | -0.207 - 0.404 | -0.030 | 0.197 | 0.879 | -0.416 - 0.356 |
| 3+sibs#II | 0.041 | 0.154 | 0.792 | | -0.262 - 0.343 | 0.031 | 0.151 | 0.835 | -0.265 - 0.328 | -0.133 | 0.163 | 0.415 | -0.452 - 0.187 |
| 3+sibs#III n-m | 0.002 | 0.144 | 0.988 | | -0.281 - 0.285 | -0.027 | 0.155 | 0.862 | -0.331 - 0.277 | -0.074 | 0.161 | 0.645 | -0.389 - 0.241 |
| 3+sibs#III m | 0.025 | 0.151 | 0.870 | | -0.271 - 0.321 | 0.011 | 0.137 | 0.938 | -0.258 - 0.279 | -0.088 | 0.147 | 0.548 | -0.376 - 0.200 |
| 3+sibs#IV | 0.012 | 0.147 | 0.934 | | -0.277 - 0.301 | 0.070 | 0.145 | 0.628 | -0.214 - 0.355 | -0.077 | 0.158 | 0.627 | -0.385 - 0.232 |
| 3+sibs#V | -0.079 | 0.186 | 0.671 | | -0.444 - 0.286 | -0.018 | 0.178 | 0.919 | -0.367 - 0.331 | -0.154 | 0.198 | 0.437 | -0.543 - 0.235 |
| 3sibs#n/a | -0.031 | 0.178 | 0.861 | | -0.381 - 0.318 | 0.036 | 0.178 | 0.838 | -0.313 - 0.385 | -0.080 | 0.191 | 0.676 | -0.455 - 0.295 |

| **1946 Age 63** | **Cancer** |  |  |  | **General Health** | | | |
| --- | --- | --- | --- | --- | --- | --- | --- | --- |
|  | **Coeff.** | **se** | **p-value** | **CI** | **Coeff.** | **se** | **p-value** | **CI** |
| Sibship size (Ref OC): 1 sibling | -0.039 | 0.080 | 0.623 | -0.197 - 0.118 | 0.008 | 0.082 | 0.927 | -0.154 - 0.169 |
| 2 siblings | -0.031 | 0.084 | 0.711 | -0.196 - 0.133 | -0.042 | 0.094 | 0.653 | -0.227 - 0.143 |
| 3+ siblings | -0.021 | 0.091 | 0.814 | -0.199 - 0.156 | 0.021 | 0.112 | 0.854 | -0.200 - 0.241 |
| Social class (ref I): II | -0.034 | 0.082 | 0.676 | -0.194 - 0.126 | -0.052 | 0.089 | 0.562 | -0.227 - 0.123 |
| III non-manual | -0.039 | 0.077 | 0.609 | -0.191 - 0.112 | -0.041 | 0.089 | 0.650 | -0.216 - 0.135 |
| III manual | -0.044 | 0.078 | 0.573 | -0.196 - 0.108 | -0.004 | 0.090 | 0.966 | -0.179 - 0.172 |
| IV | -0.016 | 0.082 | 0.849 | -0.176 - 0.145 | -0.053 | 0.092 | 0.564 | -0.234 - 0.128 |
| V | -0.050 | 0.086 | 0.561 | -0.218 - 0.118 | -0.022 | 0.112 | 0.845 | -0.242 - 0.198 |
| n/a, unemp. | 0.001 | 0.091 | 0.995 | -0.178 - 0.179 | -0.055 | 0.101 | 0.583 | -0.253 - 0.142 |
| Interactions: 1sib#II | 0.061 | 0.088 | 0.491 | -0.112 - 0.234 | 0.003 | 0.098 | 0.972 | -0.189 - 0.196 |
| 1sib#III n-m | 0.061 | 0.084 | 0.471 | -0.105 - 0.227 | 0.016 | 0.097 | 0.865 | -0.173 - 0.206 |
| 1sib#III m | 0.060 | 0.083 | 0.473 | -0.104 - 0.223 | -0.023 | 0.096 | 0.812 | -0.211 - 0.166 |
| 1sib#IV | 0.052 | 0.090 | 0.561 | -0.124 - 0.228 | -0.003 | 0.100 | 0.978 | -0.199 - 0.193 |
| 1sib#V | 0.071 | 0.102 | 0.486 | -0.129 - 0.271 | -0.006 | 0.131 | 0.961 | -0.264 - 0.251 |
| 1sib#n/a | 0.037 | 0.105 | 0.722 | -0.169 - 0.244 | -0.016 | 0.114 | 0.886 | -0.240 - 0.207 |
| 2sibs#II | 0.017 | 0.092 | 0.855 | -0.163 - 0.197 | 0.075 | 0.111 | 0.499 | -0.142 - 0.292 |
| 2sibs#III n-m | 0.047 | 0.090 | 0.603 | -0.130 - 0.223 | 0.062 | 0.111 | 0.577 | -0.155 - 0.279 |
| 2sibs#III m | 0.055 | 0.086 | 0.526 | -0.114 - 0.223 | 0.057 | 0.109 | 0.601 | -0.156 - 0.270 |
| 2sibs#IV | -0.001 | 0.091 | 0.996 | -0.179 - 0.178 | 0.087 | 0.109 | 0.426 | -0.127 - 0.301 |
| 2sibs#V | 0.102 | 0.103 | 0.322 | -0.100 - 0.304 | 0.083 | 0.141 | 0.554 | -0.193 - 0.359 |
| 2sibs#n/a | 0.001 | 0.103 | 0.992 | -0.201 - 0.203 | 0.054 | 0.127 | 0.672 | -0.196 - 0.304 |
| 3+sibs#II | 0.050 | 0.102 | 0.627 | -0.150 - 0.249 | 0.040 | 0.130 | 0.756 | -0.215 - 0.296 |
| 3+sibs#III n-m | 0.037 | 0.099 | 0.709 | -0.156 - 0.230 | 0.008 | 0.128 | 0.953 | -0.243 - 0.258 |
| 3+sibs#III m | 0.039 | 0.094 | 0.676 | -0.146 - 0.224 | 0.032 | 0.123 | 0.792 | -0.209 - 0.274 |
| 3+sibs#IV | 0.018 | 0.100 | 0.857 | -0.178 - 0.214 | 0.070 | 0.121 | 0.561 | -0.166 - 0.306 |
| 3+sibs#V | 0.069 | 0.103 | 0.500 | -0.132 - 0.271 | 0.114 | 0.140 | 0.416 | -0.160 - 0.388 |
| 3sibs#n/a | -0.005 | 0.114 | 0.964 | -0.229 - 0.219 | 0.101 | 0.139 | 0.470 | -0.173 - 0.374 |

*Notes: Coeff.= coefficient; se=Standard error; CI= 95% confidence interval; OC= only child; sibs= siblings. Outcomes are self-reported heart problems, high blood pressure (self-reported at age 55 only), high triglycerides, high Glycated haemoglobin (HbA1c), and high C-reactive protein (CRP), self-reported cancer diagnosis and self-assessed general health as fair/poor/very poor. Models run on observed cases and adjust for cohort members’ (CM) sex and birth order, maternal age at CM’s birth, maternal education, whether the CM was breastfed, paternal social class and parental separation by age 10/11 and CM’s smoking status; alcohol intake frequency; highest level of qualification; and occupation.*

**Supplementary Table S7 Summary of regression results with interaction: Coefficients for sibship size groups (reference category: Only Child) parental separation and interaction of sibship size and separation, for each health outcome, separately by cohort and age**

| **1946 Age 43** | **Heart** | | |  |  | **Hypertension** | | | | **Cancer** | | | |
| --- | --- | --- | --- | --- | --- | --- | --- | --- | --- | --- | --- | --- | --- |
|  | **Coeff.** | **se** | **p-value** | | **CI** | **Coeff.** | **se** | **p-value** | **CI** | **Coeff.** | **se** | **p-value** | **CI** |
| Sibship size (Ref OC): 1 sibling | 0.005 | 0.012 | 0.659 | | -0.018 - 0.029 | 0.001 | 0.032 | 0.976 | -0.061 - 0.063 | 0.007 | 0.011 | 0.533 | -0.014 - 0.028 |
| 2 siblings | 0.009 | 0.014 | 0.531 | | -0.019 - 0.036 | -0.007 | 0.035 | 0.837 | -0.075 - 0.061 | 0.007 | 0.012 | 0.518 | -0.015 - 0.030 |
| 3+ siblings | 0.014 | 0.014 | 0.315 | | -0.013 - 0.041 | -0.032 | 0.033 | 0.337 | -0.097 - 0.033 | -0.002 | 0.012 | 0.877 | -0.024 - 0.021 |
| Parental separation by age 11 | -0.015 | 0.027 | 0.575 | | -0.067 - 0.037 | -0.025 | 0.073 | 0.731 | -0.169 - 0.118 | 0.009 | 0.028 | 0.740 | -0.045 - 0.063 |
| 1sib#separation | 0.017 | 0.038 | 0.658 | | -0.058 - 0.091 | 0.031 | 0.091 | 0.731 | -0.147 - 0.210 | -0.005 | 0.039 | 0.902 | -0.081 - 0.072 |
| 2sibs#separation | 0.022 | 0.040 | 0.580 | | -0.057 - 0.101 | 0.073 | 0.099 | 0.464 | -0.122 - 0.268 | -0.015 | 0.034 | 0.654 | -0.081 - 0.051 |
| 3+sibs#separation | 0.022 | 0.036 | 0.544 | | -0.049 - 0.092 | 0.001 | 0.084 | 0.995 | -0.165 - 0.166 | 0.007 | 0.037 | 0.845 | -0.066 - 0.080 |

| **1958 Age 44/46** | **Heart** | |  | |  |  | **Hypertension** | | |  |  |  |  |  |  |
| --- | --- | --- | --- | --- | --- | --- | --- | --- | --- | --- | --- | --- | --- | --- | --- |
|  | **Coeff.** | **se** | | **p-value** | | **CI** | **Coeff.** | **se** | **p-value** | | **CI** |  |  |  |  |
| Sibship size (Ref OC): 1 sibling | -0.004 | 0.027 | | 0.884 | | -0.057 - 0.049 | 0.001 | 0.027 | 0.975 | | -0.052 - 0.053 |  |  |  |  |
| 2 siblings | 0.010 | 0.028 | | 0.724 | | -0.045 - 0.064 | -0.007 | 0.028 | 0.809 | | -0.062 - 0.048 |  |  |  |  |
| 3+ siblings | -0.004 | 0.028 | | 0.885 | | -0.059 - 0.051 | -0.027 | 0.030 | 0.356 | | -0.086 - 0.031 |  |  |  |  |
| Parental separation by age 11 | -0.022 | 0.021 | | 0.313 | | -0.063 - 0.020 | 0.026 | 0.085 | 0.756 | | -0.140 - 0.192 |  |  |  |  |
| 1sib#separation | 0.010 | 0.025 | | 0.684 | | -0.039 - 0.059 | -0.018 | 0.100 | 0.861 | | -0.214 - 0.179 |  |  |  |  |
| 2sibs#separation | 0.026 | 0.026 | | 0.310 | | -0.024 - 0.076 | 0.031 | 0.100 | 0.757 | | -0.166 - 0.228 |  |  |  |  |
| 3+sibs#separation | 0.039 | 0.024 | | 0.103 | | -0.008 - 0.086 | -0.004 | 0.094 | 0.964 | | -0.188 - 0.179 |  |  |  |  |
|  | **High triglycerides** | | | | | | **High glycated haemoglobin** | | | | | **High CRP** | | | |
|  | **Coeff.** | **se** | | **p-value** | | **CI** | **Coeff.** | **se** | **p-value** | | **CI** | **Coeff.** | **se** | **p-value** | **CI** |
| Sibship size (Ref OC): 1 sibling | -0.013 | 0.033 | | 0.692 | | -0.078 - 0.052 | -0.001 | 0.012 | 0.961 | | -0.024 - 0.022 | 0.086 | 0.114 | 0.451 | -0.138 - 0.309 |
| 2 siblings | -0.037 | 0.035 | | 0.290 | | -0.106 - 0.032 | -0.010 | 0.012 | 0.400 | | -0.035 - 0.014 | 0.156 | 0.117 | 0.181 | -0.073 - 0.386 |
| 3+ siblings | -0.023 | 0.037 | | 0.528 | | -0.096 - 0.049 | -0.011 | 0.013 | 0.377 | | -0.037 - 0.014 | 0.041 | 0.117 | 0.725 | -0.189 - 0.271 |
| Parental separation by age 11 | 0.093 | 0.105 | | 0.373 | | -0.112 - 0.298 | -0.034 | 0.037 | 0.357 | | -0.108 - 0.039 | 0.097 | 0.084 | 0.245 | -0.067 - 0.262 |
| 1sib#separation | -0.111 | 0.125 | | 0.375 | | -0.355 - 0.134 | 0.023 | 0.045 | 0.612 | | -0.065 - 0.110 | -0.014 | 0.100 | 0.886 | -0.210 - 0.181 |
| 2sibs#separation | 0.025 | 0.126 | | 0.840 | | -0.221 - 0.271 | 0.051 | 0.045 | 0.254 | | -0.037 - 0.139 | -0.124 | 0.100 | 0.216 | -0.321 - 0.073 |
| 3+sibs#separation | -0.113 | 0.116 | | 0.327 | | -0.340 - 0.113 | 0.055 | 0.041 | 0.179 | | -0.026 - 0.136 | -0.013 | 0.093 | 0.886 | -0.195 - 0.169 |
|  | **Cancer** | | | | | | **General Health** | | | | |  |  |  |  |
|  | **Coeff.** | **se** | | **p-value** | | **CI** | **Coeff.** | **se** | **p-value** | | **CI** |  |  |  |  |
| Sibship size (Ref OC): 1 sibling | 0.005 | 0.006 | | 0.356 | | -0.006 - 0.017 | 0.005 | 0.025 | 0.830 | | -0.043 - 0.054 |  |  |  |  |
| 2 siblings | 0.003 | 0.006 | | 0.604 | | -0.009 - 0.015 | -0.014 | 0.026 | 0.585 | | -0.065 - 0.037 |  |  |  |  |
| 3+ siblings | 0.002 | 0.006 | | 0.763 | | -0.011 - 0.014 | -0.017 | 0.028 | 0.535 | | -0.071 - 0.037 |  |  |  |  |
| Parental separation by age 11 | -0.006 | 0.018 | | 0.745 | | -0.041 - 0.029 | 0.102 | 0.078 | 0.188 | | -0.050 - 0.254 |  |  |  |  |
| 1sib#separation | -0.014 | 0.100 | | 0.886 | | -0.210 - 0.181 | -0.131 | 0.091 | 0.151 | | -0.309 - 0.048 |  |  |  |  |
| 2sibs#separation | -0.124 | 0.100 | | 0.216 | | -0.321 - 0.073 | -0.011 | 0.093 | 0.905 | | -0.193 - 0.171 |  |  |  |  |
| 3+sibs#separation | -0.013 | 0.093 | | 0.886 | | -0.195 - 0.169 | -0.096 | 0.087 | 0.269 | | -0.266 - 0.074 |  |  |  |  |

| **1970 Age 46** | **Heart** |  |  |  | **Hypertension** | | | |  |  | |  | |  |
| --- | --- | --- | --- | --- | --- | --- | --- | --- | --- | --- | --- | --- | --- | --- |
|  | **Coeff.** | **se** | **p-value** | **CI** | **Coeff.** | **se** | **p-value** | **CI** |  |  | |  | |  |
| Sibship size (Ref OC): 1 sibling | -0.006 | 0.011 | 0.565 | -0.027 - 0.015 | -0.039 | 0.029 | 0.182 | -0.095 - 0.018 |  |  | |  | |  |
| 2 siblings | -0.014 | 0.012 | 0.217 | -0.037 - 0.008 | -0.047 | 0.031 | 0.136 | -0.108 - 0.015 |  |  | |  | |  |
| 3+ siblings | -0.019 | 0.013 | 0.156 | -0.045 - 0.007 | -0.046 | 0.036 | 0.196 | -0.116 - 0.024 |  |  | |  | |  |
| Parental separation by age 10 | -0.030 | 0.021 | 0.157 | -0.070 - 0.011 | -0.042 | 0.057 | 0.458 | -0.154 - 0.069 |  |  | |  | |  |
| 1sib#separation | 0.038 | 0.023 | 0.102 | -0.008 - 0.084 | 0.047 | 0.064 | 0.466 | -0.079 - 0.172 |  |  | |  | |  |
| 2sibs#separation | 0.057 | 0.025 | 0.025 | 0.007 - 0.107 | 0.044 | 0.069 | 0.522 | -0.092 - 0.180 |  |  | |  | |  |
| 3+sibs#separation | 0.075 | 0.030 | 0.013 | 0.016 - 0.134 | 0.032 | 0.082 | 0.699 | -0.130 - 0.193 |  |  | |  | |  |
|  | **High triglycerides** | | | | **High glycated haemoglobin** | | | | **High CRP** | | | | | |
|  | **Coeff.** | **se** | **p-value** | **CI** | **Coeff.** | **se** | **p-value** | **CI** | **Coeff.** | | **se** | | **p-value** | **CI** |
| Sibship size (Ref OC): 1 sibling | -0.074 | 0.051 | 0.146 | -0.175 - 0.026 | -0.010 | 0.019 | 0.603 | -0.047 - 0.027 | -0.030 | | 0.043 | | 0.481 | -0.115 - 0.054 |
| 2 siblings | -0.063 | 0.055 | 0.255 | -0.171 - 0.045 | -0.014 | 0.020 | 0.475 | -0.054 - 0.025 | -0.045 | | 0.046 | | 0.329 | -0.136 - 0.046 |
| 3+ siblings | -0.129 | 0.062 | 0.038 | -0.251 - -0.007 | -0.032 | 0.023 | 0.158 | -0.077 - 0.013 | 0.002 | | 0.052 | | 0.969 | -0.101 - 0.105 |
| Parental separation by age 10 | -0.198 | 0.100 | 0.049 | -0.395 - -0.001 | -0.069 | 0.036 | 0.054 | -0.139 - 0.001 | -0.084 | | 0.085 | | 0.320 | -0.250 - 0.082 |
| 1sib#separation | 0.261 | 0.111 | 0.019 | 0.043 - 0.478 | 0.082 | 0.040 | 0.041 | 0.003 - 0.160 | 0.066 | | 0.094 | | 0.481 | -0.118 - 0.250 |
| 2sibs#separation | 0.126 | 0.119 | 0.288 | -0.107 - 0.359 | 0.062 | 0.044 | 0.158 | -0.024 - 0.147 | 0.028 | | 0.100 | | 0.777 | -0.168 - 0.225 |
| 3+sibs#separation | 0.132 | 0.146 | 0.368 | -0.155 - 0.419 | 0.126 | 0.052 | 0.015 | 0.025 - 0.227 | -0.087 | | 0.123 | | 0.483 | -0.328 - 0.155 |
|  | **Cancer** |  |  |  | **General Health** | | | |  |  | |  | |  |
|  | **Coeff.** | **se** | **p-value** | **CI** | **Coeff.** | **se** | **p-value** | **CI** |  |  | |  | |  |
| Sibship size (Ref OC): 1 sibling | 0.017 | 0.008 | 0.035 | 0.001 - 0.033 | 0.002 | 0.025 | 0.940 | -0.046 - 0.050 |  |  | |  | |  |
| 2 siblings | 0.020 | 0.009 | 0.027 | 0.002 - 0.037 | -0.008 | 0.027 | 0.772 | -0.060 - 0.045 |  |  | |  | |  |
| 3+ siblings | 0.027 | 0.010 | 0.008 | 0.007 - 0.046 | -0.016 | 0.030 | 0.605 | -0.075 - 0.044 |  |  | |  | |  |
| Parental separation by age 10 | -0.001 | 0.016 | 0.973 | -0.032 - 0.031 | -0.008 | 0.048 | 0.865 | -0.102 - 0.086 |  |  | |  | |  |
| 1sib#separation | 0.004 | 0.018 | 0.833 | -0.031 - 0.039 | -0.017 | 0.054 | 0.754 | -0.122 - 0.089 |  |  | |  | |  |
| 2sibs#separation | -0.007 | 0.019 | 0.737 | -0.044 - 0.031 | 0.059 | 0.058 | 0.310 | -0.055 - 0.174 |  |  | |  | |  |
| 3+sibs#separation | -0.002 | 0.023 | 0.931 | -0.047 - 0.043 | 0.005 | 0.069 | 0.945 | -0.131 - 0.140 |  |  | |  | |  |

| **1946 Age 53** | **Heart** |  |  |  | **Hypertension** | | | |
| --- | --- | --- | --- | --- | --- | --- | --- | --- |
|  | **Coeff.** | **se** | **p-value** | **CI** | **Coeff.** | **se** | **p-value** | **CI** |
| Sibship size (Ref OC): 1 sibling | 0.028 | 0.028 | 0.305 | -0.026 - 0.082 | -0.035 | 0.037 | 0.340 | -0.107 - 0.037 |
| 2 siblings | 0.040 | 0.029 | 0.164 | -0.017 - 0.097 | -0.024 | 0.038 | 0.533 | -0.098 - 0.051 |
| 3+ siblings | 0.030 | 0.026 | 0.241 | -0.020 - 0.081 | -0.051 | 0.038 | 0.185 | -0.126 - 0.024 |
| Parental separation by age 11 | 0.009 | 0.056 | 0.867 | -0.101 - 0.120 | 0.069 | 0.084 | 0.410 | -0.095 - 0.234 |
| 1sib#separation | -0.023 | 0.074 | 0.760 | -0.167 - 0.122 | -0.070 | 0.105 | 0.505 | -0.277 - 0.136 |
| 2sibs#separation | -0.028 | 0.080 | 0.725 | -0.186 - 0.129 | -0.012 | 0.112 | 0.911 | -0.232 - 0.207 |
| 3+sibs#separation | 0.006 | 0.070 | 0.932 | -0.132 - 0.144 | -0.107 | 0.102 | 0.294 | -0.307 - 0.093 |

| **1946 Age 53** | **High triglycerides** | | |  |  | **High glycated haemoglobin** | | | | **Cancer** | | | |
| --- | --- | --- | --- | --- | --- | --- | --- | --- | --- | --- | --- | --- | --- |
|  | **Coeff.** | **se** | **p-value** | | **CI** | **Coeff.** | **se** | **p-value** | **CI** | **Coeff.** | **se** | **p-value** | **CI** |
| Sibship size (Ref OC): 1 sibling | -0.036 | 0.038 | 0.333 | | -0.110 - 0.037 | -0.007 | 0.033 | 0.821 | -0.071 - 0.057 | 0.016 | 0.014 | 0.241 | -0.011 - 0.043 |
| 2 siblings | -0.017 | 0.041 | 0.678 | | -0.098 - 0.063 | -0.015 | 0.031 | 0.617 | -0.075 - 0.045 | 0.001 | 0.014 | 0.949 | -0.026 - 0.028 |
| 3+ siblings | 0.012 | 0.039 | 0.763 | | -0.066 - 0.089 | 0.027 | 0.032 | 0.398 | -0.036 - 0.090 | 0.015 | 0.013 | 0.262 | -0.011 - 0.041 |
| Parental separation by age 11 | -0.055 | 0.093 | 0.556 | | -0.238 - 0.128 | 0.005 | 0.064 | 0.943 | -0.121 - 0.130 | 0.004 | 0.031 | 0.899 | -0.056 - 0.064 |
| 1sib#separation | 0.040 | 0.119 | 0.739 | | -0.193 - 0.272 | -0.044 | 0.074 | 0.555 | -0.189 - 0.102 | 0.026 | 0.042 | 0.526 | -0.055 - 0.108 |
| 2sibs#separation | 0.044 | 0.110 | 0.687 | | -0.171 - 0.260 | -0.039 | 0.077 | 0.614 | -0.189 - 0.112 | 0.004 | 0.036 | 0.902 | -0.066 - 0.075 |
| 3+sibs#separation | 0.026 | 0.106 | 0.810 | | -0.183 - 0.234 | -0.079 | 0.079 | 0.316 | -0.235 - 0.076 | -0.001 | 0.037 | 0.983 | -0.073 - 0.071 |

| **1958 Age 55** | **Heart** |  |  |  | **Hypertension** | | | |
| --- | --- | --- | --- | --- | --- | --- | --- | --- |
|  | **Coeff.** | **se** | **p-value** | **CI** | **Coeff.** | **se** | **p-value** | **CI** |
| Sibship size (Ref OC): 1 sibling | -0.012 | 0.014 | 0.379 | -0.040 - 0.015 | -0.031 | 0.027 | 0.241 | -0.083 - 0.021 |
| 2 siblings | -0.013 | 0.015 | 0.368 | -0.042 - 0.016 | -0.040 | 0.028 | 0.160 | -0.095 - 0.016 |
| 3+ siblings | -0.003 | 0.016 | 0.830 | -0.034 - 0.027 | -0.049 | 0.030 | 0.099 | -0.107 - 0.009 |
| Parental separation by age 11 | -0.054 | 0.048 | 0.257 | -0.147 - 0.039 | -0.002 | 0.090 | 0.983 | -0.179 - 0.175 |
| 1sib#separation | 0.070 | 0.055 | 0.202 | -0.038 - 0.178 | 0.022 | 0.104 | 0.834 | -0.183 - 0.226 |
| 2sibs#separation | 0.012 | 0.056 | 0.833 | -0.098 - 0.122 | -0.013 | 0.106 | 0.903 | -0.221 - 0.195 |
| 3+sibs#separation | 0.056 | 0.053 | 0.291 | -0.048 - 0.160 | -0.021 | 0.100 | 0.832 | -0.218 - 0.175 |
|  | **Cancer** |  |  |  | **General Health** | | |  |
|  | **Coeff.** | **se** | **p-value** | **CI** | **Coeff.** | **se** | **p-value** | **CI** |
| Sibship size (Ref OC): 1 sibling | -0.010 | 0.012 | 0.410 | -0.033 - 0.013 | 0.017 | 0.024 | 0.475 | -0.029 - 0.063 |
| 2 siblings | -0.001 | 0.012 | 0.949 | -0.025 - 0.024 | 0.019 | 0.025 | 0.448 | -0.030 - 0.068 |
| 3+ siblings | -0.004 | 0.013 | 0.742 | -0.030 - 0.021 | 0.021 | 0.026 | 0.434 | -0.031 - 0.072 |
| Parental separation by age 11 | -0.039 | 0.040 | 0.324 | -0.118 - 0.039 | 0.034 | 0.080 | 0.671 | -0.123 - 0.190 |
| 1sib#separation | 0.008 | 0.046 | 0.859 | -0.082 - 0.099 | -0.059 | 0.092 | 0.522 | -0.240 - 0.122 |
| 2sibs#separation | 0.047 | 0.047 | 0.313 | -0.045 - 0.140 | -0.131 | 0.094 | 0.163 | -0.315 - 0.053 |
| 3+sibs#separation | 0.042 | 0.044 | 0.344 | -0.045 - 0.129 | -0.033 | 0.089 | 0.706 | -0.207 - 0.140 |

| **1946 Age 63** | **Heart** |  |  |  | **Hypertension** | | | |
| --- | --- | --- | --- | --- | --- | --- | --- | --- |
|  | **Coeff.** | **se** | **p-value** | **CI** | **Coeff.** | **se** | **p-value** | **CI** |
| Sibship size (Ref OC): 1 sibling | 0.025 | 0.026 | 0.336 | -0.026 - 0.076 | -0.001 | 0.049 | 0.983 | -0.098 - 0.095 |
| 2 siblings | 0.007 | 0.029 | 0.820 | -0.050 - 0.063 | -0.000 | 0.056 | 0.995 | -0.110 - 0.109 |
| 3+ siblings | 0.018 | 0.030 | 0.544 | -0.040 - 0.076 | -0.008 | 0.054 | 0.882 | -0.113 - 0.097 |
| Parental separation by age 11 | 0.017 | 0.065 | 0.790 | -0.110 - 0.145 | -0.007 | 0.090 | 0.936 | -0.183 - 0.168 |
| 1sib#separation | -0.014 | 0.079 | 0.858 | -0.169 - 0.141 | 0.037 | 0.103 | 0.719 | -0.165 - 0.239 |
| 2sibs#separation | -0.031 | 0.084 | 0.711 | -0.196 - 0.134 | -0.003 | 0.114 | 0.982 | -0.227 - 0.222 |
| 3+sibs#separation | -0.002 | 0.076 | 0.984 | -0.151 - 0.147 | 0.005 | 0.108 | 0.959 | -0.206 - 0.217 |

| **1946 Age 63** | **High triglycerides** | | |  |  | **High glycated haemoglobin** | | | | **High CRP** | | | |
| --- | --- | --- | --- | --- | --- | --- | --- | --- | --- | --- | --- | --- | --- |
|  | **Coeff.** | **se** | **p-value** | | **CI** | **Coeff.** | **se** | **p-value** | **CI** | **Coeff.** | **se** | **p-value** | **CI** |
| Sibship size (Ref OC): 1 sibling | -0.060 | 0.042 | 0.156 | | -0.143 - 0.023 | -0.042 | 0.040 | 0.291 | -0.120 - 0.036 | -0.060 | 0.043 | 0.164 | -0.145 - 0.025 |
| 2 siblings | -0.095 | 0.042 | 0.024 | | -0.178 - -0.012 | -0.035 | 0.040 | 0.374 | -0.114 - 0.043 | -0.056 | 0.045 | 0.210 | -0.144 - 0.032 |
| 3+ siblings | -0.064 | 0.041 | 0.123 | | -0.144 - 0.017 | -0.002 | 0.042 | 0.966 | -0.083 - 0.080 | -0.047 | 0.046 | 0.303 | -0.137 - 0.043 |
| Parental separation by age 11 | 0.019 | 0.086 | 0.822 | | -0.149 - 0.188 | -0.017 | 0.075 | 0.816 | -0.165 - 0.130 | -0.055 | 0.085 | 0.520 | -0.222 - 0.112 |
| 1sib#separation | -0.021 | 0.100 | 0.837 | | -0.218 - 0.176 | 0.003 | 0.092 | 0.975 | -0.177 - 0.183 | 0.019 | 0.106 | 0.860 | -0.189 - 0.226 |
| 2sibs#separation | 0.070 | 0.107 | 0.514 | | -0.140 - 0.279 | 0.022 | 0.102 | 0.829 | -0.178 - 0.222 | 0.031 | 0.103 | 0.767 | -0.171 - 0.233 |
| 3+sibs#separation | 0.010 | 0.102 | 0.925 | | -0.190 - 0.209 | 0.019 | 0.089 | 0.831 | -0.155 - 0.193 | 0.038 | 0.103 | 0.714 | -0.164 - 0.239 |

| **1946 Age 63** | **Cancer** |  |  |  | **General Health** | | | |
| --- | --- | --- | --- | --- | --- | --- | --- | --- |
|  | **Coeff.** | **se** | **p-value** | **CI** | **Coeff.** | **se** | **p-value** | **CI** |
| Sibship size (Ref OC): 1 sibling | 0.018 | 0.018 | 0.316 | -0.017 - 0.054 | 0.010 | 0.028 | 0.718 | -0.045 - 0.065 |
| 2 siblings | 0.006 | 0.020 | 0.782 | -0.034 - 0.045 | 0.030 | 0.030 | 0.322 | -0.030 - 0.090 |
| 3+ siblings | 0.016 | 0.021 | 0.435 | -0.025 - 0.057 | 0.079 | 0.032 | 0.013 | 0.016 - 0.141 |
| Parental separation by age 11 | 0.008 | 0.041 | 0.844 | -0.072 - 0.088 | 0.081 | 0.071 | 0.255 | -0.059 - 0.221 |
| 1sib#separation | -0.010 | 0.050 | 0.838 | -0.108 - 0.088 | -0.087 | 0.086 | 0.312 | -0.255 - 0.081 |
| 2sibs#separation | 0.007 | 0.055 | 0.903 | -0.101 - 0.114 | -0.050 | 0.097 | 0.603 | -0.241 - 0.140 |
| 3+sibs#separation | -0.031 | 0.046 | 0.505 | -0.122 - 0.060 | -0.025 | 0.091 | 0.785 | -0.204 - 0.154 |

*Notes: Coeff.= coefficient; se=standard error; CI= 95% confidence interval; OC= only child; sibs= siblings. Outcomes are self-reported heart problems, high blood pressure (self-reported at age 55 only), high triglycerides, high Glycated haemoglobin (HbA1c), and high C-reactive protein (CRP), self-reported cancer diagnosis and self-assessed general health as fair/poor/very poor. Models run on observed cases and adjust for cohort members’ (CM) sex and birth order, maternal age at CM’s birth, maternal education, whether the CM was breastfed, paternal social class and parental separation by age 10/11 and CM’s smoking status; alcohol intake frequency; highest level of qualification; and occupation.*

1. Graham JW. Missing Data Analysis: Making It Work in the Real World. Annual review of psychology 2009; 60: 549-76.; Jeličić H, Phelps E, Lerner RM. Use of Missing Data Methods in Longitudinal Studies: The Persistence of Bad Practices in Developmental Psychology. Developmental psychology 2009; 45: 1195-9.; Mostafa T, Narayanan M, Pongiglione B, et al. Missing at random assumption made more plausible: evidence from the 1958 British birth cohort. Journal of clinical epidemiology 2021; 136: 44-54. [↑](#footnote-ref-1)
